# Genome-Wide Analysis of a *TaLEA*-Introduced Transgenic *Populus simonii × Populus nigra* Dwarf Mutant

**DOI:** 10.3390/ijms13032744

**Published:** 2012-03-01

**Authors:** Hong-Mei Yuan, Su Chen, Lin Lin, Rui Wei, Hui-Yu Li, Gui-Feng Liu, Jing Jiang

**Affiliations:** 1 State Key Laboratory of Forest Genetics and Tree Breeding, Northeast Forestry University, 26 Hexing Road, Harbin 150040, China; E-Mails: chensunefu@163.com (S.C.); yuanhm1979@163.com (H.-M.Y.); linlin198212@126.com (L.L.); wrrachel@yahoo.com.cn (R.W.); 44919635@qq.com (H.-Y.L.); liuguifeng@126.com (G.-F.L.); 2 College of Life Science, Daqing Normal University, Daqing 163712, China

**Keywords:** dwarf, mutant, poplar, microarrays, *AP2*, *RAV*

## Abstract

A dwarf mutant (*dwf1*) was obtained among 15 transgenic lines, when *TaLEA* (*Tamarix androssowii* late embryogenesis abundant gene) was introduced into *Populus simonii × Populus nigra* by *Agrobacterium tumefaciens*-mediated transformation. Under the same growth conditions, *dwf1* height was significantly reduced compared with the wild type and the other transgenic lines. Because only one transgenic line (*dwf1*) displayed the dwarf phenotype, we considered that T-DNA insertion sites may play a role in the mutant formation. The mechanisms underlying this effect were investigated using TAIL-PCR (thermal asymmetric interlaced PCR) and microarrays methods. According to the TAIL-PCR results, two flanking sequences located on chromosome IV and VIII respectively, were cloned. The results indicated the integration of two independent T-DNA copies. We searched for the potential genes near to the T-DNA insertions. The nearest gene was a putative poplar *AP2* transcription factor (GI: 224073210). Expression analysis showed that *AP2* was up-regulated in *dwf1* compared with the wild type and the other transgenic lines. According to the microarrays results, a total of 537 genes involved in hydrolase, kinase and transcription factor activities, as well as protein and nucleotide binding, showed significant alterations in gene expression. These genes were expressed in more than 60 metabolic pathways, including starch, sucrose, galactose and glycerolipid metabolism and phenylpropanoids and flavonoid biosyntheses. Our transcriptome and T-DNA insertion sites analyses might provide some useful insights into the dwarf mutant formation.

## 1. Introduction

In our previous study, a *Tamarix androssowii LEA* gene (*lea* IV; DQ663481; *TaLEA*) was introduced into *Populus simonii × Populus nigra* to improve salt tolerance [1]. Among these transgenic lines, only one, *dwf1*, exhibited a dwarf phenotype. This indicated that the T-DNA insertion might be responsible for this mutation. The insertions can be determined using TAIL-PCR (thermal asymmetric interlaced PCR). TAIL-PCR is performed with an arbitrary genomic primer and a T-DNA-specific primer on DNA templates. Such priming creates both specific and non-specific products, whose relative amplification efficiencies can be thermally controlled. TAIL-PCR has become an extremely valuable and versatile tool in all research involving recovery of unknown genomic sequences adjacent to known sequences and it has been utilized in functional genomics [2,3].

Dwarf mutants in plants are crucial for elucidating the regulatory mechanisms of plant growth and development. Various factors are responsible for dwarfism in plants, but the phytohormones gibberellin (GA), brassinosteroids (BRs) and auxin are the most intensely studied factors that determine plant height [4,5]. Dwarfism usually results from mutations in genes that regulate the biosyntheses of these plant hormones or are involved in their signaling transduction pathways.

GAs are a large family of endogenous growth regulators in higher plants that are involved in a wide range of processes in plant growth and development, such as seed germination, stem elongation, leaf expansion, pollen maturation and flower induction. Recently, numerous GA-related dwarf mutants, including GA-deficient or GA-insensitive dwarf mutants, have been identified that display typical dwarfism characteristics, in addition, have wide leaf blades and dark green leaves [6,7].

BRs are plant steroids that are essential for normal growth and development and these are involved in germination, cell elongation, photomorphogenic responses and male fertility. Therefore, BR biosynthesis, transduction and response mutants have seriously deficient phenotypes in terms of plant growth and development. Genes encoding 5-alpha-reductase and cytochrome P450 enzymes have been cloned and shown to be involved in BR biosynthesis and metabolism [8–10].

Recently, studies on BRASSINOSTEROID-INSENSITIVE1 (BRI1, a receptor of BR) and several other important components involved in BR signaling have provided much insight into many important components in plant development. BRI1 is a plasma membrane-localized protein. Its extracellular domain contains 21 tandem *N*-terminal leucine-rich repeats (LRRs), a 70-amino acid island domain and four additional LRRs that precede the transmembrane domain. BRI1 is considered to be the most important regulator in the BR pathways, as it plays critical roles in the direct binding of BR and subsequent signaling processes [11–14].

The T-DNA insertion sits in *dwf1* might be responsible for the dwarf mutant formation, considering that only one transgenic line exhibiting dwarf phenotype. TAIL-PCR and microarray analysis were performed to clone the flanking sequences of T-DNA insertions and further analyze the transcriptome profile of *dwf1*. Our analyses indicated that a putative poplar *AP2* transcript factor nearby one T-DNA insertion sit was up-regulated. More than 500 genes were differently expressed in *dwf1* compared with the wild type plant, including plant hormone receptors and 27 transcript factors. The up-regulated *AP2* and the differently expressed genes in *dwf1* might give some useful insights for further analyze the dwarf mechanism, All of these results may prove to be potential candidates for global genetic engineering of development regulation in poplar.

## 2. Results and Discussion

### 2.1. Characterization of the *Dwf1* Mutant

**Figure 1:**
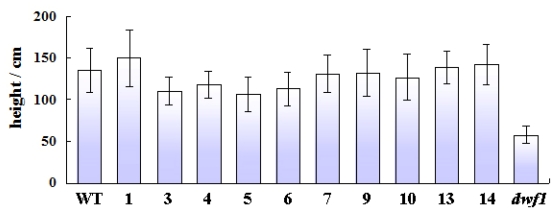
Mean height of the one-year-old transgenic lines in Qiqihar city plantation. WT: wild type; 1, 3, 4, 5, 6, 7, 9, 10, 13 and 14: transgenic lines; *dwf1*: dwarf mutant. Mean height of each was derived from 50 individual.

**Figure 2:**
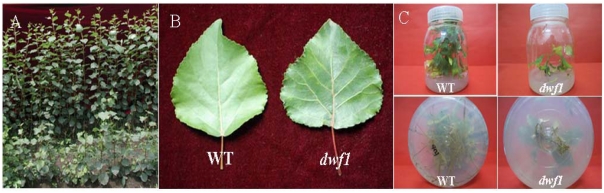
Phenotypic characterization of *dwf1* and wild type under the same growth conditions. (**A**) Propagation of *dwf1* (front row) and wild type (back row); (**B**) Leaves of *dwf1* and the wild type; (**C**) Tissue culture seedlings of *dwf1* and wild type. The *dwf1* mutant displayed lower root biomass, fewer lateral roots, crinkly and leathery leaves, dark green leaf blades and deep red petioles, in comparison to the wild type and the other transgenic lines.

Among 15 independent *TaLEA* transgenic lines, one line showed significantly reduced height in all plants, and this line was named *dwf1*. In addition, the mutant showed lower root biomass, fewer lateral roots, crinkly and leathery leaves, dark green leaf blades and deep red petioles, in comparison to the wild type and the other transgenic lines (Table 1). Chlorophyll content of *dwf1* was higher compared to the wild type and the transgenic line 5 and 6. *Dwf1* exhibited a less leaf length-width ratio. These characteristics indicated that *dwf1* had multiple morphological defects (Figures 1 and 2).

### 2.2. Analyses of T-DNA Flanking Sequences

**Figure 3:**
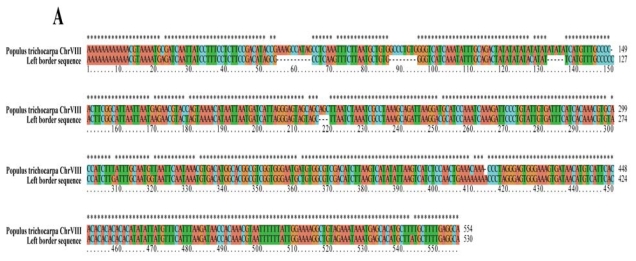

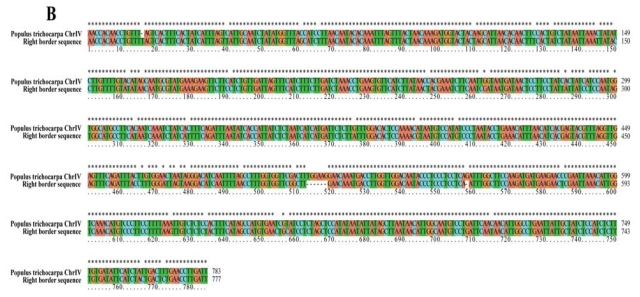
Alignment analysis of the right and left border sequence against NCBI polar genome database. (**A**) right border sequence; (**B**) left border sequence.

TAIL-PCR method was employed to determine the T-DNA insertion sites in *dwf1*. We obtained an 847 bp right border and a 698 bp left border flanking sequence (Table S1). Sequence alignments of the sequences against the pROK binary vectors sequence and the NCBI poplar genome database showed that the right and the left border mapped to different chromosomes (Figure 3). The right border was located at Chr IV (10,570,141 bp), while the left border was located at Chr VIII (7,459,761 bp). The results indicated that there was more than one independent T-DNA copy.

**Figure 4:**
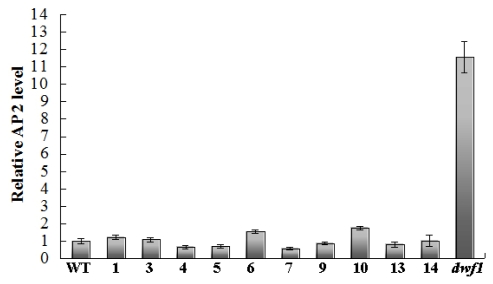
Relative *AP2* expression level. The *AP2* gene showed a high expression level in *dwf1* compared to the wild type and the other transgenic lines. QRT-PCR data were normalized using poplar *actin* (GI: 224116599) and *tublin* (GI: 224054577) genes. Standard deviations were derived from three replicates performed for each experiment.

NCBI Map Viewer was used to search for potential expressed genes adjacent to the insertions [15]. Only a putative *AP2* transcription factor (GI: 224073210) was found 3 kb downstream the insertion site of the left border (Table 2). Quantitative RT-PCR analysis showed that *AP2* was up-regulated in *dwf1* compared with the wild type and the other transgenic lines (Figure 4).

**Figure 5:**
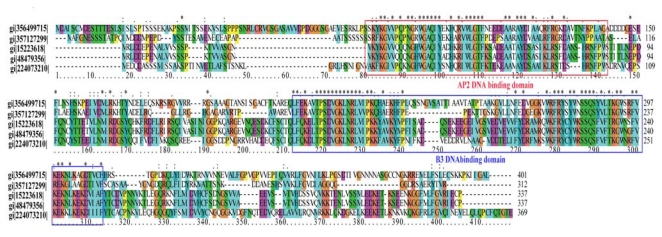
Sequence alignment and characterization analyses of *AP2* genes. The *AP2* gene encodes a protein of 369 amino acids containing two DNA-binding domains, an AP2 DNA-binding domain in the *N*-terminal region and a B3 domain in the *C*-terminal region. gi|224073210|: *Populus trichocarpa* AP2 domain-containing transcription factor; gi|15223618|: *Arabidopsis thaliana* RAV-like factor; gi|48479356|: *Arabidopsis thaliana* putative AP2/EREBP transcription factor; gi|356499715|: *Glycine max* PREDICTED: AP2/ERF and B3 domain-containing transcription repressor TEM1-like; gi|357127299|: *Brachypodium distachyon* PREDICTED: putative AP2/ERF and B3 domain-containing protein Os01g0140700-like.

Sequence analysis revealed that the *AP2* gene encodes a protein of 369 amino acids containing two DNA-binding domains, an AP2 and a B3 DNA-binding domain (Figure 5).

The *AP2/ERF* superfamily is defined by the AP2/ERF domain, which consists of about 60 to 70 amino acids that are involved in DNA-binding. Members of this superfamily can be divided further into three families. The AP2 family proteins contain two repeat AP2/ERF domains, the ethylene response factor (ERF) family proteins contain a single AP2/ERF domain, while the RAV family proteins contain a B3 domain and a single AP2/ERF domain [16,17]. The AP2/ERF proteins are involved in a variety of biological processes related to plant growth and development, as well as various responses to environmental stimuli [18–20].

Recently, members of the *RAV* family were reported to be involved in plant responses to ethylene and brassinosteroids (BRs) [21,22]. Transgenic tobacco overexpressing the *GmRAV* (*Glycine max RAV*) gene had slower plant growth rates, reduced root elongation, delayed flowering times, reduced photosynthetic rates and reduced chlorophyll contents in leaves [23]. The RAV1 transcription factor also positively regulates leaf senescence in Arabidopsis [24].

It has been proven that *RAV* genes play an important role in maintaining brassinosteroid homeostasis in higher plants. Brassinosteroids (BRs) are plant steroid hormones that are perceived by the cell surface receptor kinase Brassinosteroid Insensitive1 (BRI1). In rice *RAVL1* regulates the expression of the BR receptor [25]. Furthermore, *RAVL1* is also required for the expression of the BR biosynthetic genes. According to the microarray analysis results, putative BRI1-associated receptor kinase 1 precursor genes (PtpAffx.35408.1.S1_at, PtpAffx.48409.1.S1_at) were up-regulated in *dwf1*. All of these results indicated that there might be some relationship between the overexpression of *AP2* and the dwarf phenotype of *dwf1*. However, the functional role of *AP2* in dwarf formation still needs a further confirmation.

### 2.3. Genome-Wide mRNA Expression Analysis in the *Dwf1* Mutant

**Figure 6:**
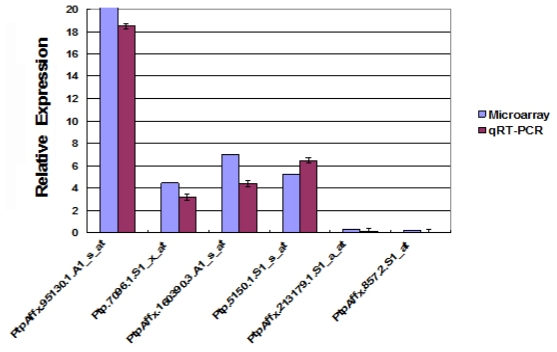
Verification of differential gene expression by quantitative real time-PCR compared with microarrays data. Six genes were selected at random for qRT-PCR assays. Expression of these six genes obtained by qRT-PCR fitted in well with the pattern of microarrays results. QRT-PCR data were normalized using poplar *actin* (GI: 224116599) and *tublin* (GI: 224054577) genes. Standard deviations were derived from three replicates performed for each experiment.

To analyze further the molecular mechanisms responsible for the *dwf1* phenotype, the mRNA profile of *dwf1* and the wild type were compared using an Affymetrix whole-genome microarray chip. Compared with the wild type, 537 genes were differentially expressed, including 257 down-regulated genes and 280 up-regulated genes in the *dwf1* mutant (Tables S. 2,3). To validate these microarrays data, six genes were selected at random for quantitative RT-PCR assays. Each of these six genes displayed an expression pattern consistent with the microarray analysis results (Figure 6). Therefore, further analyses focused on these 537 differentially expressed genes.

### 2.4. Functional Classification of Differentially Expressed Genes

**Figure 7:**
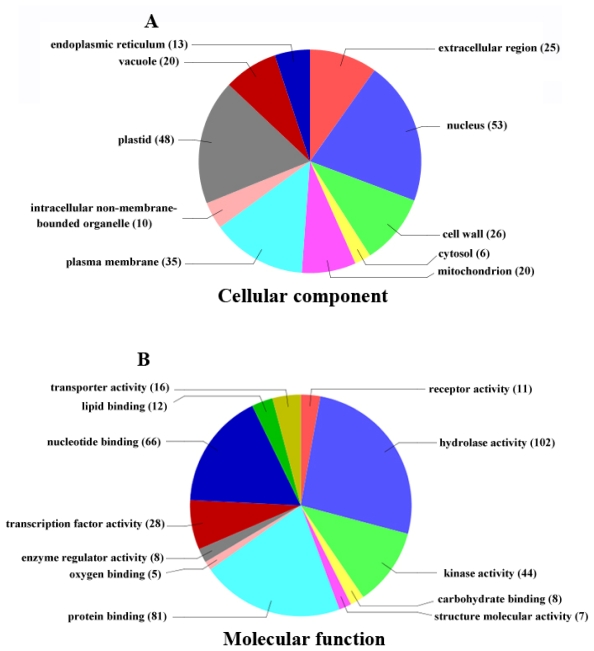

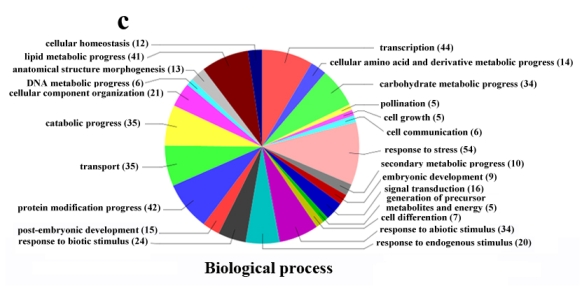
Functional categorization of the genes that are significantly differentially expressed in the *dwf1* mutant and the wild type.

To evaluate the potential functions of genes with significant transcriptional changes between the *dwf1* mutant and the wild type, gene ontology (GO) categories were assigned to 443 genes of the 537 genes based on the plant GO slim provided by blast2GO [26]. The categorization of differentially expressed genes according to their cellular component, molecular function and biological process is shown in Figure 7. According to cellular component, the analysis revealed a high percentage of genes corresponding to the nucleus, plastid, plasma membrane, cell wall, extracellular region, mitochondrion and vacuole. Based on molecular function, the most represented GO terms were hydrolase activity, protein binding, nucleotide binding, kinase activity and transcription factor activity. Differentially expressed genes related to more than 20 biological processes, including responses to stress, transcription, protein modification processes, lipid and carbohydrate metabolism, transport, catabolic processes, responses to abiotic and biotic stimuli, responses to endogenous stimuli and cellular component organization.

### 2.5. Biological Pathway Analyses According to the Kyoto Encyclopedia of Genes and Genomes (KEGG)

KEGG pathway analysis was used to identify the biological pathways of the genes that were significantly differentially expressed. More than 60 different metabolic pathways were involved; some were consistent with biological processes revealed already by GO analysis. The most represented pathways included pathways involved with the biosyntheses of secondary metabolites, flavonoids, phenylpropanoids, starch, sucrose, terpenoids, steroids, plant hormones and alkaloids derived from the shikimate pathway, and the metabolism of galactose and glycerolipids. Many of these metabolic pathways are related to plant hormones, which are responsible for controlling plant growth and development.

### 2.6. Genes Related to Plant Hormones

Many plant hormone related genes showed differential expression patterns in *dwf1*. These genes included plant hormone receptors, such as F-box, LRR receptor-like and G-proteins, ubiquitin, cytochrome P450 genes, and plant hormone response genes (Table 3).

**Figure 8:**
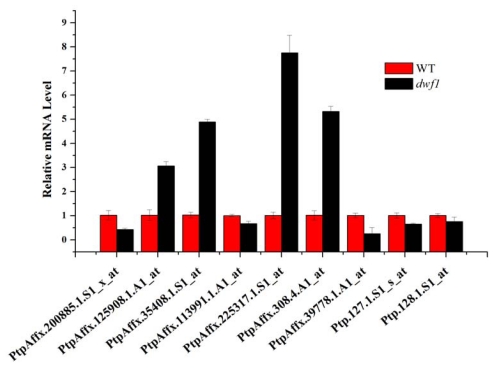
Relative expression level of plant hormone related genes. PtpAffx.200885.1.S1_x_at and PtpAffx.125908.1.A1_at are F-box family protein; PtpAffx.35408.1.S1_at is BRASSINOSTEROID INSENSITIVE 1-associated receptor kinase 1 precursor; PtpAffx.113991.1.A1_at is E2 ubiquitin-conjugating enzyme UBC10; PtpAffx.225317.1.S1_at is Cytochrome P450-like; PtpAffx.308.4.A1_at is sterol delta-7 reductase DWF5; PtpAffx.39778.1.A1_at, Ptp.127.1.S1_s_at and Ptp.128.1.S1_at are aux/IAA protein.

In plants, nearly every aspect of the plant’s life is regulated by plant hormones. Central to comprehending hormonal control of plant growth and development is the understanding of how the hormones are perceived. In the past few years, some plant hormone receptors have been identified. F-box proteins such as TIR1 [27] and AFBs are receptors of auxin. Auxin plays a vital role in regulating plant growth and development [28,29]. Jasmonic acid shares the same receptors with auxin [30]. Leucine-rich repeat receptor-like kinases, BRI1, are the receptors of Brassinosteroids [14]. In this study, three potential F-box family proteins PtpAffx.200885.1.S1_x_at, PtpAffx.45694.1.S1_at and PtpAffx.125908.1.A1_at showed differential expression levels (Figure 8).

Plant cytochromes P450 (CYPs) are involved in a wide range of biosynthetic reactions, leading to various fatty acid conjugates, plant hormones, defensive compounds, or medically important drugs. Terpenoids, which represent the largest class of characterized natural plant compounds, are often substrates for plant CYPs. In this study more than 10 cytochrome P450s showed different expression patterns.

The ubiquitin-proteasome system (UPS) is involved in nearly every aspect of plant growth and development. Ubiquitin is attached to target proteins through the action of three enzymes known as E1, E2, and E3. Ubiquitination is a recurring theme in plant signal transduction. E3 ubiquitin ligases in particularly participate in hormone perception [31–34]. In this study 5 ubiquitins and 2 ubiquitins ligases showed different expression patterns. All of these might affect the perception and the transduction of plant hormones, and plants’ growth will be abnormal if they are sensitive or insensitive to plant hormones.

### 2.7. Transcription Factors with Differential Expression Patterns

Transcription factors regulate gene expression at the level of transcription via the recognition of promoter elements. In this study 27 differentially regulated genes encoded putative transcription factors, including 23 up-regulated and 4 down-regulated genes (Table 4). More than half of the transcription factors were from WRKY, AP2, and ERF families, while the others belonged to various families, including heat shock transcription factors and myb families. WRKY transcription factors are one of the largest families of transcriptional regulators in plants and form integral parts of signaling webs that modulate many plant processes [35–38].

### 2.8. Transcript Profile Analysis of the Plant Hormones Related Genes and Transcript Factors in Other Transgenic Lines by Using Digital Gene Expression Method

Using the microarrays method we obtained differently expressed genes in *dwf1* compared with wild type plants. However, misregulation could be due to the *LEA* transgene on the T-DNA. We performed a Digital Gene Expression (DGE) to analysis the gene expression patterns in transgenic line 5 (XL-5), 6 (XL-6) with normal phenotype, and *dwf1*. Specifically, we analyzed the expression patterns of the plant hormone related genes and transcription factors in transgenic line 5, 6, and *dwf1*. According to the digital gene expression (DGE) results (Table 5), nearly all of these differentially expressed genes, especially plant hormone related, between *dwf1* and WT did not change expression level in XL-5 and XL-6 as compared to the wild type. The results indicated that *dwf1* showed a unique gene expression profile compared to wild type and the other transgenic lines.

## 3. Experimental Section

### 3.1. Plant Materials and Transgene

A F_1_ hybrid of *Populus simonii × Populus nigra* was generated in this study. The transgenic poplar lines were produced as described previously [39]. For co-cultivation, *Agrobacterium tumefaciens* strain was incubated in liquid LB medium at 28 °C with constant shaking (250 rpm) until the culture reached an optical density of about 0.6 at 600 nm. The *A. tumefaciens* culture was then diluted with one volume of liquid MH medium. Leaves of *Populus simonii × Populus nigra* excised from plantlets cultured *in vitro* were cut into discs and cultured in the dark for 2–3 days on MH medium containing 0.5 mg/L 6-benzylaminopurine (6-BA), 0.05 mg/L 1-naphthaleneacetic acid (NAA), and 0.8% (w/v) agar. The leaf discs were then dipped in the diluted Agrobacterium culture for about 2–5 min and cultured on co-cultivation medium. After co-cultivation in the dark for 2 days, the leaf discs were transferred to MH medium containing 0.5 mg/L 6-BA, 0.05 mg/L NAA, 200 mg/L Cefotaxime, 50 mg/L kanamycin and 0.8% (w/v) agar and cultured in the light for 7 days. The regenerated shoots were individually removed from the callus and transferred to solid MH medium containing 0.05 mg/L 6-BA, 0.01 mg/L NAA, 50 mg/L kanamycin, and 0.8% (w/v) agar. Regenerated shoots were transferred to rooting medium (MH containing 0.2 mg/L indolebutyric acid, sucrose 15 g/L, and 50 mg/L kanamycin).

The seedlings of wild type and *dwf1* were transferred to a greenhouse and planted in a mixture of turfy peat and sand (2:1 v/v) with 75% relative humidity and a constant temperature of 24 °C. Young leaves were harvested from three independent biological replicates with five plants per replicate. Leaves were immersed immediately in liquid nitrogen and stored at −70 °C for subsequent DNA and RNA extractions.

### 3.2. RNA Isolation and cDNA Synthesis

Total RNA was prepared using the sodium dodecyl sulfate (SDS) method [40]. RNA integrity was examined by electrophoresis on a 1% formaldehyde denaturing gel. The samples with bright bands corresponding to ribosomal 28S and 18S RNA (with a ratio of intensity of ca. 1.5 to 2.5:1) were used for microarray analyses and qRT-PCR. First strand cDNA was generated from 1 μg RNA using PrimeScript^®^ RT reagent kit (Perfect Real Time; TaKaRa, Japan) with oligo-dT primers and according to the manufacturer’s protocol.

### 3.3. Quantitative Real-Time PCR

Quantitative real-time PCR (Q-PCR) was conducted using SYBR Green dye (SYBR^®^ Green Realtime PCR Master Mix-Plus-, Code No. QPK-212). PCR was conducted according to the manual. Parallel reactions using *actin* (GI:224116599) and *tublin* (GI:224054577) genes were performed to normalize the amount of template cDNA. The protocol of real-time PCR was as follows: initiation with a 2 min denaturation at 94 °C, followed by 45 cycles of amplification with 10 s of denaturation at 94 °C, 15 s of annealing at 60 °C, 30 s of extension at 72 °C and reading the plate for fluorescence data collection at 75 °C. A melting curve was performed from 75 to 95 °C to check the specificity of the amplified product. Primer sequences for the real-time PCR assay are listed in Table S4. Three PCR replicates were performed for each RNA sample.

### 3.4. DNA Isolation and Identification of T-DNA Insertion Site

Genomic DNA was isolated from the leaves of the *dwf1* and wild type plants using a modified CTAB method as described by Porebski [41]. The T-DNA insertion site in the *dwf1* mutant was determined by TAIL-PCR [2]. The primers GSPL1, GSPL2 and GSPL3 (Table S5) were used for the cloning of the flanking sequence of the left border, while the primers GSPR1, GSPR2 and GSPR3 (Table S4) were used for the right border. The arbitrary degenerate primer was provided by the Genome Walking kit (Takara Genome Walking Kit, code: D316, Japan). TAIL-PCR amplification was performed according to Sissions’ procedure [42]. After three rounds of PCR reactions, the products were separated by electrophoresis on 1.5% agarose gels. The resulting bands were purified using the Aqua-SPIN Gel Extraction Ace kit (Watson Biotechnologies, China), cloned using the pMD18-T cloning kit (TaKaRa, Japan), and sequenced (Invitrogen, China). The NCBI popular genome database was used to analyze the flanking sequences.

### 3.5. Affymetrix Microarray Analysis Experiment and Microarrays Data Analysis

Three sets of biological replicates were collected independently and a total of six gene chips were analyzed. For the microarray analysis experiment, an aliquot of 2 μg of total RNA was used to synthesize double-stranded cDNA. Then, biotin-tagged cRNA was produced using the MessageAmpTMII aRNA Amplification kit. The biotin-tagged cRNA was fragmented into strands of 35 to 200 bases in length according to the Affymetrix protocol. The fragmented cRNA was hybridized to Affymetrix poplar genome AFF-900728 that contains 56,055 transcripts. Hybridization was performed at 45 °C with rotation for 16 h (Affymetrix GeneChip Hybridization Oven 640). The GeneChip arrays were washed and then stained (streptavidin phycoerythrin) on an Affymetrix Fluidics Station 450, followed by scanning on a GeneChip Scanner 3000. The hybridization data were analyzed using GeneChip Operating software (GCOS 1.4). The scanned images were assessed first by visual inspection, and then analyzed to generate raw data files (saved as CEL files) using the default settings of GCOS 1.4. A global scaling procedure was performed to normalize the different arrays using the dChip software.

Statistical analyses were performed to identify differently expressed genes between *dwf1* and wild type using SAM (significance analysis of microarrays) method described previously [43]. In this study we used the fold change ≥ 2 or ≤ 0.5 and *q*-value ≤ 0.05 as the threshold to judge the significance of gene expression.

### 3.6. DGE Analysis

DGE is a method that generates absolute gene expression measurements. DGE libraries were prepared using Illumina Gene Expression Sample Preparation Kit according to the manufacturer’s instructions. Briefly, 6 μg total RNA was used for mRNA capture with magnetic Oligo(dT) beads. The first and second cDNA strands were synthesized using Oligo(dT) primers. The 5′ ends of tags were generated by Endonuclease *N*laIII, which recognizes and cuts off the CATG sites. The fragments apart from the 3′ cDNA fragments connected to Oligo(dT) beads were washed away and the Illumina adaptor 1 was ligated to the sticky 5′ end of the digested bead-bound cDNA fragments. The junction of Illumina adaptor 1 and CATG site is the recognition site of *M*meI, which is a type of endonuclease with separate recognition sites and digestion sites. It cuts at 17bp downstream of the CATG site, producing tags with adaptor 1. After removing 3′ fragments with magnetic beads precipitation, Illumina adaptor 2 was ligated to the 3′ ends of tags, acquiring tags with different adaptors of both ends to form a tag library. After 15 cycles of linear PCR amplification, 105 bp fragments were purified by 6% TBE PAGE gel electrophoresis. After denaturation, the single-chain molecules were fixed onto the Illumina Sequencing Chip (flowcell). Each molecule grows into a single-molecule cluster sequencing template through *in situ* amplification. Then four types of nucleotides labeled by four colors were added, and sequencing using the method of sequencing by synthesis (SBS) were performed. Raw sequence reads were filtered by the Illumina pipeline. Low-quality sequences such the 3′ adaptor sequence, Tags which were too long or too short, Tags with unknown sequences and Tags with only 1 copy were removed. The remaining high quality sequences (Clean Tags) were mapped to Poplar reference sequences [44]. Clean Tags mapped to poplar genome sequences from multiple genes were filed. The gene expression was normalized to transcripts per million clean tags (TPM).

### 3.7. Chlorophyll Content

A commercial chlorophyll meter SPAD-502 was used to estimate the leaves chlorophyll content. 6 independent plants were selected for each transgenic line and WT.

## 4. Conclusions

In order to improve salt resistance, transgenic poplar plants overexpressing *TaLEA* were obtained [1]. Among the transgenic lines, only one transgenic line (*dwf1*) showed a dwarf phenotype. Considering that, we analyzed the T-DNA insertion sites in *dwf1* using TAIL-PCR. Results showed that the right and left border located at different chromosomes. This indicated that there was more than one T-DNA copy in *dwf1*. We analyzed the potential genes near to the T-DNA insertion sites. A putative poplar *AP2* transcription factor was found. QRT-PCR analysis indicated that the *AP2* gene was up-regulated in *dwf1* compared with the wild type plant and the other transgenic lines. Bioinformatics analysis indicated that the *AP2* gene belonged to *RAV* family of *AP2* superfamily. *RAV* family genes were reported to play an import role in maintaining plant hormone homeostasis [20–24]. To identify genes that were responsible for the mutant formation, we further analyzed the *dwf1* transcript profile using microarray analysis. More than 500 differentially expressed genes were identified. Many plant hormone related genes were affected. These genes included plant hormone receptors and plant hormone response genes. 27 transcript factors including *AP2* and *WRKY* were especially affected. The differentially expressed genes might offer some clues for further analysis of the dwarf formation. In addition, these genes may prove to be potential candidates for global genetic engineering of development regulation in poplar. However, all of these results still need farther tests and verification.

## Supplementary Information



## Figures and Tables

**Table 1:** Chlorophyll content, leaf area, leaf length-width ratio, and root length of WT, XL-5, XL-6 and *dwf1* (*P* < 0.5, *N* = 50).

Transgenic Line	Chlorophyll Content (SPAD)	Leaf Area (cm^2^)	Length/Width	Root Length (cm)
WT	32.14 ± 4.04 a	43.79 ± 13.21 a	1.11 ± 0.14 a	4.08 ± 0.35 a
XL-5	32.66 ± 3.71 a	42.27 ± 9.22 a	1.05 ± 0.13 a	4.10 ± 0.47 a
XL-6	31.53 ± 5.74 a	29.85 ± 5.71 b	1.04 ± 0.13 a	4.17 ± 0.41 a
*dwf1*	37.15 ± 4.24 b	39.54 ± 10.49 a	0.90 ± 0.08 b	1.95 ± 0.51 b

**Table 2:** NCBI map viewer results of T-DNA insertion regions.

Border Sequence		Start	Stop	Description
Right border	T-DNA insertion sit	10,569,359 bp	10,570,141 bp	Chr IV
	region displayed	10,566,600 bp	10,573,600 bp	no putative genes
Left border	T-DNA insertion sit	7,459,761 bp	7,460,314 bp	Chr VIII
	region displayed	7,456,200 bp	7,463,200 bp	putative *AP2* transcription factor

**Table 3:** Genes related to plant hormones.

Probe Set ID	Description	Fold Change (*dwf1*/WT)
Receptors Related		

PtpAffx.225074.1.S1_at	Receptor-related protein kinase-like	13.8804
PtpAffx.75698.1.A1_at	Receptor-like serine/threonine kinase	10.9634
PtpAffx.35408.1.S1_at	BRASSINOSTEROID INSENSITIVE 1-associated receptor kinase 1 precursor, putative	7.4219
PtpAffx.224953.1.S1_x_at	Leucine-rich repeat receptor-like protein kinase 1	5.951
PtpAffx.18756.1.S1_a_at	Receptor serine/threonine kinase, putative	5.1724
Ptp.1239.2.S1_at	Vacuolar sorting receptor, putative	4.4382
PtpAffx.223006.1.S1_s_at	leucine-rich repeat receptor-like protein kinase	4.2346
PtpAffx.225656.1.S1_at	Receptor-like protein kinase homolog RK20-1	3.9744
PtpAffx.225480.1.S1_at	(Receptor Like Protein 19); kinase/ protein binding	3.9717
PtpAffx.26522.1.S1_at	Stress-induced receptor-like kinase	3.329
PtpAffx.48409.1.S1_at	BRASSINOSTEROID INSENSITIVE 1-associated receptor kinase 1 precursor	2.8082
PtpAffx.225585.1.S1_at	Receptor-like protein kinase 4, putative (RLK4)	2.3771
PtpAffx.50721.1.A1_s_at	Receptor serine-threonine protein kinase, putative	0.3798
PtpAffx.222905.1.S1_s_at	Leucine-rich repeat receptor-like protein kinase	0.3393
PtpAffx.200885.1.S1_x_at	F-box family protein	0.4794
PtpAffx.200885.1.S1_at	F-box family protein	0.4765
PtpAffx.45694.1.S1_at	F-box family protein	0.3875
PtpAffx.125908.1.A1_at	F-box protein family	2.2595

Cytochrome P450 genes		

PtpAffx.225317.1.S1_at	Cytochrome P450-like	7.2083
PtpAffx.224780.1.S1_s_at	Putative cytochrome P450	3.1479
PtpAffx.225200.1.S1_at	Cytochrome P450 family protein	2.6336
PtpAffx.124621.1.S1_s_at	Cytochrome P450	2.3608
Ptp.2894.1.A1_at	Cytochrome P450	2.1906
PtpAffx.203255.1.S1_s_at	Cytochrome P450, putative	0.4878
PtpAffx.220980.1.S1_s_at	Cytochrome P450 82C1-soybean	0.4874
Ptp.962.1.S1_s_at	Cytochrome P450 monooxygenase CYP77A3v2	0.4822
PtpAffx.209025.1.S1_at	Putative cytochrome P450	0.4497
PtpAffx.224872.1.S1_s_at	Cytochrome P450	0.3524
PtpAffx.158051.1.A1_at	Cytochrome P450	0.2578

Ubiquitins		

PtpAffx.20179.1.A1_at	Ubiquitin	2.3644
PtpAffx.20179.3.A1_at	Ubiquitin	2.3096
PtpAffx.20179.2.S1_at	Ubiquitin	2.0378
Ptp.7320.1.S1_a_at	Ubiquitin, putative	2.0015
PtpAffx.2814.1.A1_at	Ubiquitin-protein ligase, putative	0.4885
Ptp.3476.1.S1_s_at	Ubiquitin	0.4401
PtpAffx.113991.1.A1_at	E2 ubiquitin-conjugating enzyme UBC10	0.4293

Plant hormone response genes		

PtpAffx.113518.1.S1_at	Dormancy/auxin associated protein-related	3.2637
PtpAffx.218532.1.S1_s_at	Auxin-responsive GH3 family protein	2.3382
PtpAffx.72392.1.A1_at	GH3 family protein	2.2979
Ptp.128.1.S1_at	Aux/IAA protein	0.4928
PtpAffx.123395.1.S1_at	Auxin-induced protein	0.4888
PtpAffx.204268.1.S1_at	Auxin-induced protein 15A	0.4348
PtpAffx.39778.1.A1_at	Aux/IAA protein	0.4174
Ptp.127.1.S1_s_at	Aux/IAA protein	0.4027

**Table 4:** Genes related to transcription factors.

Probe Set ID	Description	Fold Change (*dwf1*/WT)
PtpAffx.37401.1.A1_s_at	Basic-leucine zipper (bZIP) transcription factor	18.4804
PtpAffx.107622.1.A1_at	WRKY transcription factor, putative	17.1165
PtpAffx.205219.1.S1_at	WRKY family transcription factor	11.0952
PtpAffx.211278.1.S1_at	WRKY family transcription factor	10.6904
Ptp.5995.1.S1_at	WRKY transcription factor, putative	5.4609
PtpAffx.101694.1.A1_at	WRKY transcription factor, putative	2.6628
PtpAffx.5224.1.A1_at	WRKY transcription factor 2	2.551
PtpAffx.22176.1.A1_at	Transcriptional factor B3	2.4954
PtpAffx.26228.1.S1_at	AP2/ERF domain-containing transcription factor	2.4415
PtpAffx.37783.1.A1_s_at	WRKY transcription factor, putative	2.4322
PtpAffx.10330.2.S1_a_at	putative zinc finger (B-box type) family protein	2.4221
PtpAffx.202579.1.S1_x_at	myb family transcription factor (MYB114)	2.4031
PtpAffx.16157.1.S1_s_at	Transcription factor GT-3b	2.3955
Ptp.4550.1.S1_at	Transcription factor LHY	2.3908
PtpAffx.132628.1.S1_s_at	Putative WRKY transcription factor 30	2.2339
PtpAffx.34370.1.S1_at	Transcription factor, putative	2.1851
PtpAffx.221169.1.S1_at	Transcriptional factor B3 family protein	2.1293
PtpAffx.214377.1.S1_s_at	AP2-like ethylene-responsive transcription factor	2.1219
PtpAffx.219808.1.S1_at	Homeodomain transcription factor ATHB-51	2.1169
PtpAffx.152367.1.S1_s_at	WRKY transcription factor, putative	2.0796
PtpAffx.34524.3.A1_a_at	AP2 domain-containing transcription factor	2.0643
PtpAffx.200385.1.S1_at	Ethylene-responsive transcription factor CRF2	2.0245
PtpAffx.1802.2.S1_at	Heat shock transcription factor (HSF)	2.0112
PtpAffx.147430.1.A1_at	AP2/ERF domain-containing transcription factor	0.4931
PtpAffx.200077.1.S1_s_at	AP2-like ethylene-responsive transcription factor	0.4527
PtpAffx.68429.2.S1_at	WRKY transcription factor, putative	0.4489

**Table 5:** Expression level of hormone related genes and transcription factors in XL-5, XL-6, and *dwf1*. XL-5, and XL-6: transgenic lines; *dwf1*: dwarf mutant.

Probe Set ID	Fold Change (microarrays) *dwf1*/WT	Fold Change (DGE)		

		XL-5/WT	XL-6/WT	*dwf1*/WT
Hormones related				

PtpAffx.35408.1.S1_at	7.4219	1.02	1.07	13.81
PtpAffx.223006.1.S1_s_at	4.2346	1.34	1.76	3.49
PtpAffx.222905.1.S1_s_at	0.3393	1.34	1.06	0.66
PtpAffx.200885.1.S1_x_at	0.4794	1.02	0.98	0.87
PtpAffx.200885.1.S1_at	0.4765	0.81	0.78	0.32
PtpAffx.45694.1.S1_at	0.3875	1.21	1.34	0.65
PtpAffx.224780.1.S1_s_at	3.1479	0.82	1.21	4.51
PtpAffx.225317.1.S1_at	7.2083	0.91	0.75	5.55
PtpAffx.20179.1.A1_at	2.3644	1.71	1.52	3.12
Ptp.127.1.S1_s_at	0.4027	1.02	1.31	0.22
PtpAffx.34524.3.A1_a_at	2.0643	1.77	0.95	3.09
PtpAffx.5224.1.A1_at	2.551	1.55	1.78	1.87
PtpAffx.200077.1.S1_s_at	0.4527	1.57	1.88	0.58
PtpAffx.214377.1.S1_s_at	2.1219	0.53	0.78	4.59
