# Supplementary material for: Genome-Wide Analysis of a TaLEA-Introduced Transgenic Populus simonii × Populus nigra Dwarf Mutant
Source: Int J Mol Sci. 2012 Mar 1;13(3):2744–62. doi: 10.3390/ijms13032744 (PMC3317382; doi:10.3390/ijms13032744)
Supplement: Supplementary file 1 [file ijms-13-02744-s001.pdf]

Supplementary Information

Table S1. Sequences of right border and left border.

	Sequence
Right border	AACCACAACCTGTTTTAGTCACTTTCACTATCATTTAGTTATTGCAATCTATATGGTTTAGCATCTTTAACAATACACAAA<br>TTTAGTTTACTAACAAAGATGGTACTACTAGCATTAACACAACTTTCACTATCTATAATTAAATTATACCTTGTTTTGTATA<br>TAACAATGCGTATGAAAGAAGTTCTTCCTCTGTTGATTAGTTTCATCTTTCTTGATCTAAACCTGAAGTGTTCATCTTAT<br>AACTACGAAATCTTCAATCGATAATGATAACTCCTTCCTATTATTATCCTCCAATAGTGGCATGCCTTCATAATCAAATCT<br>ATCATTTTCAGATTTAATATCACCATTATCTCTAATCATCATGATTCTCTTATTTGGACACTCCAAAACGTAATGTCCATGT<br>CCCTAATACCTGAAACATTTAACATCACGAGTACGTTTAGGTTGAGTTTCAGATTTACCTTTGGGATTAGTAAGGACAT<br>CAATTTTAACCTTTGGTGGTTCGGCTTGAACAAATGACCTTGGTTGGACAATACCCTCCCTCCTCAATTTGGCTTCCAA<br>GATGATGAAGAACTCGAATTAAACATTGGTCAAACATGTCCCTTCCTTTTAAGTTGTCTCTCTACTTTCATAGCCATGTG<br>AACTGCATCCTCTAGCTCCATATAATATTATAGCTTAATAACATTGGCAATGTCCTGATTCAATAACATTGGCCTGAATTA<br>TTGCTATCTCCATCTCTTTGTGATATTCATCTACTGACTCTGAACCTTGATTCAAACACTGATAGTTTAAACTGAAGGCG<br>GGAAACGACAATCTGATCCTGAGCGGAGAATTAAGGGAGTCA
Left border	ACCGCTTGCTGCAACTCTCTCAGGGCCAGGCAGTGAAGGGCAATCAGCTGTTGCCCGTCTCACTGGTGAAAAGAAA<br>AACCACCCCAGTACATTAAAAACGTCCGCAATGTGTTATTAAGTTGTCTAAGCGTCAATTTGTTTACACCACAATATATC<br>CTATCAACATCCAAAAAAAAAAAAACGTAAAATGAGATCAATTATCCTTTCCTCTTCCGACATAGCGCCTCAAGTTTCTT<br>AATGCTGTGGGGTCATCAAATATTTGCAGACTATATATATACATATTCATGTTTGCCCCCACTTCGGCATTAATTAATAAG<br>AACGTACTAGTAAAACATAATTAATGATCATTAGGGAGTAGTAGCTTAATCTAAATCGCCTAAAGCAGATTAAGGACGC<br>ATCCAAATCAAAGATTCCCTGTATTGTGATTTCATCACAAACGTGTACCATCTTGATTTGCAATGGTAATTCAATAAATG<br>TGACATGGCACGGCGTCGGTGGGAATGCTGTGGCGTCGACATCTTAAGTCATATATTAAGTCATCTCCAACTGAAAAA<br>AAACCCTAGGGAGTGGGAAAGTGATAACATGTCATTCACACACACACACACATATATTATGTTTCATTTAAGATAACCA<br>CAAACGTAATTTTTTATTGGAAAAGGCTGTAGAAATAAATGAGCACATGCTTATGCTTTTGAGGCA

Table S2. Up-regulated genes in Affymetrix microarray analysis.

Probe Set ID	Protein Acc. No.	Description	Fold Change (*dwf1*/WT)
PtpAffx.5388.2.A1_s_at	A9PE69	predicted protein [*Populus trichocarpa*]	29.5643
PtpAffx.215262.1.S1_s_at	Q9SUB6	basic helix-loop-helix (bHLH) family protein [*Arabidopsis thaliana*]	23.0943
PtpAffx.953.2.A1_at	B9NF46	predicted protein [*Populus trichocarpa*]	21.0908
PtpAffx.95130.1.A1_s_at	B9HBG1	ATEXPB4, putative [*Ricinus communis*]	20.7922
PtpAffx.165.1.S1_at	Q0WLS9	Nucleoid DNA-binding protein cnd41-like protein[*Arabidopsis thaliana*]	19.6213
PtpAffx.101048.1.A1_at	B9HJ22	hypothetical protein [*Vitis vinifera*]	19.3471
PtpAffx.37401.1.A1_s_at	B9GK20	basic-leucine zipper (bZIP) transcription factor [*Dictyostelium discoideum* AX4]	18.4804
PtpAffx.107622.1.A1_at	B9HDJ4	WRKY transcription factor, putative [*Ricinus communis*]	17.1165
PtpAffx.211308.1.S1_s_at	Q7X9I9	caffeic acid *O*-methyltransferase. [*Rosa chinensis* var. spontanea]	16.0388
PtpAffx.211583.1.S1_at	P23472	Hevamine-A [*Hevea brasiliensis*]	14.6828
PtpAffx.225074.1.S1_at	Q9XGZ2	receptor-related protein kinase - like [*Arabidopsis thaliana*]	13.8804
PtpAffx.151041.1.S1_s_at	B9H2I8	senescence-specific cysteine protease [*Nicotiana tabacum*]	13.7083
Ptp.1553.1.S1_a_at	B9MV18	predicted protein [*Populus trichocarpa*]	11.9363
PtpAffx.75508.1.A1_at	B9IHR2	VQ motif family protein [*Zea mays*]	11.5506
PtpAffx.10106.1.S1_at	B9GX94	CXE carboxylesterase [*Malus pumila*]	11.4806
PtpAffx.205219.1.S1_at	Q93WU9	WRKY family transcription factor [*Arabidopsis thaliana*]	11.0952
PtpAffx.75698.1.A1_at	B9IH68	receptor-like serine/threonine kinase [*Glycine max*]	10.9634
PtpAffx.211278.1.S1_at	Q9LY00	WRKY family transcription factor	10.6904
PtpAffx.23427.1.S1_at	B9HKX9	glutathione *S*-transferase GST 18 [*Populus alba* × *Populus tremula var. glandulosa*]	10.5221
PtpAffx.200308.1.S1_at	P35748	myosin heavy chain [*Oryctolagus cuniculus*]	10.4238
PtpAffx.37785.1.A1_s_at	B9GJ60	1-aminocyclopropane-1-carboxylate oxidase, putative [*Ricinus communis*]	9.0271
PtpAffx.202401.1.S1_s_at	Q9FNZ5	NPR1/NIM1-interacting protein 1 (NIMIN-1) [*Arabidopsis thaliana*]	8.9526
PtpAffx.224953.1.S1_s_at	NP_849538.2	leucine-rich repeat family protein/protein kinase family protein [*Arabidopsis thaliana*]	8.6793
PtpAffx.32356.3.S1_at	Q9M5I9	beta-1,3 glucanase [*Populus tremula* × *Populus alba*]	8.5691
PtpAffx.82458.1.A1_s_at	B9HNV6	1-acylglycerol-3-phosphate acyltransferase, putative [*Ricinus communis*]	8.1951
PtpAffx.210308.1.S1_at	P09886	Small heat shock protein, chloroplast precursor[*Pisum sativum* (pea)]	7.8248
Ptp.4882.2.S1_at	B9HSZ5	protein disulfide isomerase [*Quercus suber*]	7.812
PtpAffx.225490.1.S1_s_at	Q9C6A6	Disease resistance protein, putative; [*Arabidopsis thaliana*]	7.6622

Table S2. *Cont.*

PtpAffx.210610.1.S1_at	Q9SKB2	leucine-rich repeat transmembrane protein kinase, putative [*Arabidopsis thaliana*]	7.6442
PtpAffx.102182.1.A1_at	B9GJ60	alkaloid *o*-methyltransferase related [*Populus trichocarpa*]	7.5229
PtpAffx.35408.1.S1_at	B9HEA2	BRASSINOSTEROID INSENSITIVE 1-associated receptor kinase 1 precursor, putative [*Ricinus communis*]	7.4219
PtpAffx.225317.1.S1_at	Q9LVY7	Cytochrome P450-like [*Arabidopsis thaliana*]	7.2083
PtpAffx.207588.1.S1_s_at	O23507	Proteinase like protein[*Arabidopsis thaliana*]	7.1934
PtpAffx.160390.3.A1_s_at	B9I292	1-aminocyclopropane-1-carboxylate oxidase, putative [*Ricinus communis*]	6.9324
PtpAffx.160390.3.A1_at	Q9FLV0	Flavanone 3-hydroxylase-like protein[*Arabidopsis thaliana*]	6.8563
Ptp.4542.1.S1_at	B9I8S5	0similar to chitinase [*Vitis vinifera*]	6.618
PtpAffx.224400.1.S1_at	O03982	heavy-metal-associated domain-containing protein [*Arabidopsis thaliana*]	6.2032
PtpAffx.64603.1.S1_at	B9HYK4	Acidic endochitinase SE2 precursor, putative [*Ricinus communis*]	5.9736
PtpAffx.224953.1.S1_x_at	BAC87845	leucine-rich repeat receptor-like protein kinase 1[*Populus nigra*]	5.951
PtpAffx.18581.1.A1_at	B9N086	NIMIN2c protein [*Nicotiana tabacum*]	5.7996
PtpAffx.85937.1.A1_s_at	A9PG28	Glucan endo-1,3-beta-glucosidase precursor, putative [*Ricinus communis*]	5.7221
Ptp.4747.1.A1_x_at	Q9M069	glycosyl hydrolase family 17 protein [*Arabidopsis thaliana*]	5.6271
PtpAffx.46526.1.S1_at	B9H1P6	NAC domain protein, IPR003441 [*Populus trichocarpa*]	5.5484
Ptp.5995.1.S1_at	B9HDB0	WRKY transcription factor, putative [*Ricinus communis*]	5.4609
Ptp.5150.1.S1_s_at	B9HEH9	Miraculin precursor, putative [*Ricinus communis*]	5.237
Ptp.4222.1.S1_at	AAV34889.2	osmotin-like [*Theobroma cacao*]	5.1754
PtpAffx.18756.1.S1_a_at	B9NFR1	receptor serine/threonine kinase, putative [*Ricinus communis*]；	5.1724
PtpAffx.1235.1.A1_at	B9H1N8	lysine/histidine transporter [*Populus trichocarpa*]	5.1166
PtpAffx.160390.2.A1_at	B9IDF7	1-aminocyclopropane-1-carboxylate oxidase, putative [*Ricinus communis*]	4.8349
PtpAffx.18784.1.A1_at	B9RTC0	Major allergen Pru ar, putative [*Ricinus communis*]	4.7312
PtpAffx.138853.1.A1_at	B9R8N8	serine/threonine-protein kinase cx32, putative [*Ricinus communis*]	4.6826
PtpAffx.211922.1.S1_at	Q9FLM5	*N*-hydroxycinnamoyl/benzoyltransferase-like protein [*Arabidopsis thaliana*]	4.5745
PtpAffx.2173.1.S1_at	B9SXZ7	Disease resistance response protein, putative [*Ricinus communis*]	4.5128
PtpAffx.5388.1.A1_at	A5Z1X8	lipid transfer protein [*Populus alba* × *Populus tremula var. glandulosa*]	4.5103
PtpAffx.223051.1.S1_s_at	Q06086	Tyrosine decarboxylase 2 [*Petroselinum crispum*]	4.4722
Ptp.7096.1.S1_x_at	Q5CCP3	glutathione *S*-transferase GST 18 [*Populus alba* × *Populus tremula var. glandulosa*]	4.457

Table S2. *Cont.*

Ptp.1239.2.S1_at	B9GN10	vacuolar sorting receptor, putative[*Arabidopsis thaliana*]	4.4382
PtpAffx.68101.1.S1_s_at	B9SH99	nucleoredoxin, putative [*Ricinus communis*]	4.378
PtpAffx.308.4.A1_at	Q2QCX7	sterol delta-7 reductase DWF5 [*Gossypium hirsutum*]	4.3057
Ptp.7515.1.S1_s_at	Q9FX13	ankyrin repeat family protein[*Arabidopsis thaliana*]	4.2854
PtpAffx.223006.1.S1_s_at	Q8RWZ9	Leucine-rich repeat receptor-like protein kinase (Putative receptor serine/threonine kinase) [*Arabidopsis thaliana*]	4.2346
PtpAffx.204446.1.S1_at	Q9ZVC6	heat shock family protein [*Arabidopsis thaliana*]	4.1978
PtpAffx.125484.1.S1_at	A9QVH3	heat shock protein [*Ammopiptanthus mongolicus*]	4.1857
PtpAffx.224390.1.S1_s_at	Q8VYG0	serine/threonine protein kinase family protein [*Arabidopsis thaliana*]	4.0904
PtpAffx.212299.1.S1_at	Q8W4T9	Taxane 13-alpha-hydroxylase [*Taxus cuspidata*]	4.0569
PtpAffx.10261.1.S1_at	O65659	VQ motif-containing protein [*Arabidopsis thaliana*]	4.0242
PtpAffx.43231.1.A1_x_at	B9T3K4	glutathione s-transferase, putative [*Ricinus communis*]	4.0163
PtpAffx.225656.1.S1_at	Q9XED4	receptor-like protein kinase homolog RK20-1. [*Phaseolus vulgaris*]	3.9744
PtpAffx.225480.1.S1_at	Q9ZUK3	(Receptor Like Protein 19); kinase/ protein binding [*Arabidopsis thaliana*]	3.9717
Ptp.4014.1.S1_s_at	B9SWM7	heat-shock protein, putative [*Ricinus communis*]	3.9618
PtpAffx.3177.1.A1_at	B9SIY5	zinc finger protein, putative [*Ricinus communis*]	3.9402
PtpAffx.31297.1.A1_at	B9RH77	ATP binding protein, putative [*Ricinus communis*]	3.925
PtpAffx.249.570.S1_a_at	Q9SSV4	myo-inositol-1-phosphate synthase [*Nicotiana paniculata*]	3.9188
PtpAffx.148444.1.S1_at	Q9M4G9	putative ripening-related protein [*Vitis vinifera*]	3.8933
PtpAffx.8720.2.S1_at	B9S8H1	zinc finger protein, putative [*Ricinus communis*]	3.8278
PtpAffx.85855.1.S1_at	B9RT30	Cullin-1, putative [*Ricinus communis*]	3.8202
PtpAffx.3597.1.S1_s_at	B9RYU1	cystathionine gamma-synthase, putative [*Ricinus communis*]	3.6597
PtpAffx.41858.1.A1_s_at	B9RBD1	DNA binding protein, putative [*Ricinus communis*]	3.6107
PtpAffx.18527.1.A1_at	B9RVT4	Protein SIS1, putative [*Ricinus communis*]	3.5402
PtpAffx.223050.1.S1_s_at	P54768	tyrosine/dopa decarboxylase Papaver somniferum (opium poppy)	3.4967
PtpAffx.203591.1.S1_s_at	Q9SGU9	Similar to O-succinylhomoserine sulfhydrylase[*Arabidopsis thaliana*]	3.4865
PtpAffx.200248.1.S1_at	Q94AG2	leucine-rich repeat family protein/protein kinase family protein [*Arabidopsis thaliana*]	3.4779
Ptp.4901.1.S1_s_at	B9RRD9	pentatricopeptide repeat-containing protein, putative [*Ricinus communis*]	3.4771
PtpAffx.210188.1.S1_s_at	Q9ZUW8	DC1 domain-containing protein [*Arabidopsis thaliana*]	3.4721

Table S2. *Cont.*

PtpAffx.212146.1.S1_at	B9RUJ7	ATP binding protein, putative [*Ricinus communis*]	3.4627
PtpAffx.43322.5.A1_s_at	B9IQA6	Metalloendoproteinase 1 precursor, putative [*Ricinus communis*]	3.4626
PtpAffx.214214.1.S1_at	Q3E8P7	AtGUS3 (*Arabidopsis thaliana* glucuronidase 3); beta-glucuronidase[*Arabidopsis thaliana*]	3.3771
PtpAffx.2212.1.A1_at	B9IPB5	ATP binding protein, putative [*Ricinus communis*]	3.3649
PtpAffx.222739.1.S1_s_at	Q8GYH9	protein kinase-related [*Arabidopsis thaliana*]	3.3399
PtpAffx.26522.1.S1_at	C6FF75	stress-induced receptor-like kinase [*Glycine max*]	3.329
PtpAffx.69188.2.A1_s_at	B9S6A0	ATP binding protein, putative [*Ricinus communis*]	3.3275
PtpAffx.206771.1.S1_at	Q8L828	coatomer protein complex, subunit beta 2 (beta prime), putative	3.2937
Ptp.2730.1.S1_at	B9IQ33	calreticulin, putative [*Ricinus communis*]	3.277
PtpAffx.133484.1.A1_at	Q9ZUW8	DC1 domain-containing protein [*Arabidopsis thaliana*]	3.2667
PtpAffx.113518.1.S1_at	Q9SYG3	dormancy/auxin associated protein-related [*Arabidopsis thaliana*]	3.2637
PtpAffx.35380.1.S1_a_at	B9I1Z6	fad NAD binding oxidoreductases, putative [*Ricinus communis*]	3.2069
PtpAffx.208930.1.S1_s_at	Q9C6K9	protein kinase family protein [*Arabidopsis thaliana*]	3.1937
PtpAffx.249.474.S1_at	B9T7K3	myo-inositol-1 phosphate synthase, putative [*Ricinus communis*]	3.1842
PtpAffx.116237.1.A1_a_at	B9T780	ring finger protein, putative [*Ricinus communis*]	3.1777
Ptp.5280.1.S1_at	B9RM48	calreticulin, putative [*Ricinus communis*]...	3.1598
PtpAffx.224780.1.S1_s_at	O81971	putative cytochrome P450 [*Glycine max*]	3.1479
Ptp.1501.1.A1_s_at	B9S6B6	Mitochondrial chaperone BCS1, putative [*Ricinus communis*]	3.1433
Ptp.7306.2.S1_at	B9HT83	nucleoredoxin, putative [*Ricinus communis*	3.14
PtpAffx.210421.1.S1_at	Q9FKL6	calmodulin-binding protein [*Arabidopsis thaliana*]	3.1274
PtpAffx.82275.1.A1_at	Q8HQ02	ATP synthase subunit a [*Thrips imaginis*]	3.1195
PtpAffx.159602.1.A1_at	B9P872	predicted protein [*Populus trichocarpa*]	3.1169
PtpAffx.71047.1.S1_at	B9T6S0	Metalloendoproteinase 1 precursor, putative [*Ricinus communis*]	3.0866
PtpAffx.98587.2.S1_at	B9IJZ4	calcium ion binding protein, putative [*Ricinus communis*]	3.0822
PtpAffx.54628.1.S1_at	B9IGP7	Cationic peroxidase 1 precursor, putative [*Ricinus communis*]	3.059
PtpAffx.222254.1.S1_at	O22243	zinc finger (CCCH type) helicase family protein [*Arabidopsis thaliana*]	3.055
PtpAffx.225021.1.S1_at	Q9SK82	putative UDP-glucose glucosyltransferase[*Arabidopsis thaliana*]	3.0391
PtpAffx.202805.1.S1_at	Q9SQV0	GNS1/SUR4 membrane family protein [*Arabidopsis thaliana*]	3.037

Table S2. *Cont.*

Ptp.3289.1.A1_at	B9H1T7	ATP binding protein, putative [*Ricinus communis*]	3.0337
PtpAffx.214555.1.S1_at	Q3E9A4	catalytic [*Arabidopsis thaliana*]	3.0086
PtpAffx.201546.1.S1_at	A4FVS9	protein kinase, putative [*Arabidopsis thaliana*]	2.983
PtpAffx.153843.1.A1_at	B9GRS9	NAC domain protein, IPR003441 [*Populus trichocarpa*]	2.9603
PtpAffx.205847.1.S1_at	Q8GT74	zinc finger (C3HC4-type RING finger) family protein [*Arabidopsis thaliana*]	2.9127
PtpAffx.110.1.S1_at	B9RX83	serine-threonine protein kinase, plant-type, putative [*Ricinus communis*]	2.8845
Ptp.5778.1.S1_at	P32289	GLNA_VIGAC RecName: Full=Glutamine synthetase nodule isozyme	2.8673
PtpAffx.7040.1.S1_at	Q9LJB7	zinc finger (B-box type) family protein [*Arabidopsis thaliana*]	2.8661
Ptp.3690.1.S1_at	B9IIS2	NAC domain protein, IPR003441 [*Populus trichocarpa*]	2.8609
PtpAffx.29235.1.A1_at	B9RNR9	Aspartic proteinase nepenthesin-2 precursor, putative [*Ricinus communis*]	2.8583
PtpAffx.6353.1.S1_at	B9RCK8	Disease resistance response protein, putative [*Ricinus communis*]	2.8556
Ptp.5070.1.S1_s_at	B9S7V4	STS14 protein precursor, putative [*Ricinus communis*]	2.8534
PtpAffx.491.1.S1_s_at	A1E2A9	small molecular heat shock protein 17.5 [*Nelumbo nucifera*]	2.8409
PtpAffx.36446.1.A1_s_at	B7U488	myo-inositol 1-phosphate synthase type 4 [*Brassica napus*]	2.835
PtpAffx.48409.1.S1_at	B9RIJ2	BRASSINOSTEROID INSENSITIVE 1-associated receptor kinase 1 precursor, putative [*Ricinus communis*]	2.8082
PtpAffx.206099.1.S1_at	Q944B1	beta-1,3-glucanase. [*Prunus persica*]	2.8036
PtpAffx.221983.1.S1_at	Q9LP11	AAA-type ATPase family protein [*Arabidopsis thaliana*]	2.7869
PtpAffx.205724.1.S1_s_at	AAA92677	calmodulin-like protein. [*Pisum sativum*]	2.7844
Ptp.6165.1.S1_at	B9N9K4	glutaredoxin C9 [*Populus trichocarpa*]	2.7451
PtpAffx.204864.1.S1_at	Q9FJ76	long-chain-alcohol O-fatty-acyltransferase family protein/wax synthase family protein [*Arabidopsis thaliana*]	2.7313
PtpAffx.205148.1.S1_at	Q9S745	PAD4 (PHYTOALEXIN DEFICIENT 4); lipase/ protein binding/triacylglycerol lipase [*Arabidopsis thaliana*]	2.725
PtpAffx.218944.1.S1_s_at	Q9ZP55	dynamin family protein[*Arabidopsis thaliana*]	2.7237
PtpAffx.204741.1.S1_at	Q8LCZ8	leaf senescence-associated protein (SAG101) [*Arabidopsis thaliana*]	2.721
PtpAffx.146630.1.A1_at	B9GI76	NAC domain protein, IPR003441 [*Populus trichocarpa*]	2.7189
PtpAffx.91912.1.S1_at	B9IDH5	acidic ribosomal protein P1a-like [*Solanum tuberosum*]	2.7086
PtpAffx.224458.1.S1_s_at	Q3E978	serine carboxypeptidase S28 family protein [*Arabidopsis thaliana*]	2.6894

Table S2. *Cont.*

PtpAffx.212818.1.S1_s_at	Q64K29	(−)-germacrene D synthase. [*Populus balsamifera* subsp. trichocarpa × *Populus deltoides*]	2.6838
Ptp.2360.1.S1_s_at	B9RE27	structural constituent of cell wall, putative [*Ricinus communis*]	2.6662
Ptp.4101.1.S1_s_at	O49821	glutathione transferase [*Carica papaya*]	2.6646
PtpAffx.101694.1.A1_at	B9T535	WRKY transcription factor, putative [*Ricinus communis*]	2.6628
PtpAffx.15983.1.S1_s_at	Q6ZXI2	putative wall-associated kinase [*Populus* × *canadensis*]	2.6498
PtpAffx.220821.1.S1_s_at	Q9SA25	protein kinase family protein [*Arabidopsis thaliana*]	2.6469
Ptp.6145.1.S1_at	C6ZRZ6	disease resistance protein [*Glycine max*]	2.6445
PtpAffx.225200.1.S1_at	Q9STI1	cytochrome P450 family protein [*Arabidopsis thaliana*]	2.6336
Ptp.7527.1.S1_s_at	B9S8N9	zinc finger protein, putative [*Ricinus communis*]	2.6285
PtpAffx.225559.1.S1_s_at	Q9LMP1	WAK2; ATP binding/calcium ion binding/protein kinase/ protein serine/threonine kinase [*Arabidopsis thaliana*]	2.6244
Ptp.2360.1.S1_at	Q6DMM3	Cel9B [*Populus tremula* × *Populus tremuloides*]	2.6148
PtpAffx.224972.1.S1_at	Q9SXB8	S-locus lectin protein kinase family protein [*Arabidopsis thaliana*]	2.6089
PtpAffx.203366.1.S1_at	B9DG31	CTP synthase, putative/UTP-ammonia ligase, putative [*Arabidopsis thaliana*]	2.5658
Ptp.417.1.S1_s_at	A8WCU4	glutamine synthetase [*Gossypium herbaceum*]	2.5658
PtpAffx.36103.1.S1_a_at	B9RAL6	Anthranilate N-benzoyltransferase protein, putative [*Ricinus communis*]	2.5638
PtpAffx.5224.1.A1_at	Q0GGW9	WRKY transcription factor 2 [*Gossypium hirsutum*]	2.551
PtpAffx.156067.1.A1_s_at	B9RDJ6	Adenosine 3'-phospho 5'-phosphosulfate transporter, putative [*Ricinus communis*]	2.5438
PtpAffx.22176.1.A1_at	Q2HUJ5	Transcriptional factor B3 [*Medicago truncatula*]	2.4954
Ptp.732.2.S1_at	O64743	MEE23 (MATERNAL EFFECT EMBRYO ARREST 23); FAD binding/catalytic/ electron carrier/ oxidoreductase [*Arabidopsis thaliana*]	2.4497
PtpAffx.26228.1.S1_at	B9GS58	AP2/ERF domain-containing transcription factor [*Populus trichocarpa*]	2.4415
PtpAffx.18969.1.A1_at	C0KHK6	non-specific lipid-transfer protein type 2 [*Tamarix hispida*]	2.4367
PtpAffx.104298.1.S1_at	B9RFU7	interferon-induced GTP-binding protein mx, putative [*Ricinus communis*]	2.4339
PtpAffx.37783.1.A1_s_at	B9SRT4	WRKY transcription factor, putative [*Ricinus communis*]	2.4322
PtpAffx.10330.2.S1_a_at	B9SIH7	transcription factor, putativezinc finger (B-box type) family protein [*Ricinus communis*]zinc finger (B-box type) family protein	2.4221
Ptp.5106.1.S1_s_at	Q653F1	putative blight-associated protein p12 precursor [*Oryza sativa Japonica* Group]	2.4186

Table S2. *Cont.*

PtpAffx.3203.5.S1_at	B9R8A7	Endoplasmin, putative [*Ricinus communis*]	2.4179
PtpAffx.142812.1.S1_s_at	B9REF8	beta-glucosidase, putative [*Ricinus communis*]	2.4178
PtpAffx.152256.1.S1_at	B9SXA3	chaperone protein DNAj, putative [*Ricinus communis*]	2.4086
PtpAffx.85677.1.S1_at	B9H498	white-brown-complex ABC transporter family [*Populus trichocarpa*]	2.4066
Ptp.5321.1.S1_s_at	B9RIF9	Lichenase precursor, putative [*Ricinus communis*]	2.4063
PtpAffx.202579.1.S1_x_at	Q9FNV8	myb family transcription factor (MYB114) [*Arabidopsis thaliana*]	2.4031
PtpAffx.16157.1.S1_s_at	Q84QS1	transcription factor GT-3b [*Arabidopsis thaliana*]	2.3955
PtpAffx.216301.1.S1_s_at	Q84NI7	alkaline alpha galactosidase I. [*Cucumis melo*]	2.3908
Ptp.4550.1.S1_at	B7X9P2	transcription factor LHY [*Populus nigra*]	2.3908
PtpAffx.225407.1.S1_s_at	Q9M0X5	protein kinase family protein [*Arabidopsis thaliana*]	2.3865
PtpAffx.5430.5.S1_at	B9R8R1	Glucan endo-1,3-beta-glucosidase precursor, putative [*Ricinus communis*]	2.3818
PtpAffx.225585.1.S1_at	Q8GYA4	receptor-like protein kinase 4, putative (RLK4) [*Arabidopsis thaliana*]	2.3771
PtpAffx.23373.3.A1_at	B9SC93	nicotinate phosphoribosyltransferase, putative [*Ricinus communis*]	2.375
PtpAffx.200274.1.S1_at	Q9LPD9	DNA replication licensing factor, putative [*Arabidopsis thaliana*]	2.373
PtpAffx.49686.1.S1_at	B9RUF0	extensin, proline-rich protein, putative [*Ricinus communis*]	2.3704
PtpAffx.27740.1.S1_at	B9RXT6	ATP binding protein, putative [*Ricinus communis*]	2.3665
PtpAffx.20179.1.A1_at	Q94813	ubiquitin [*Tetrahymena pyriformis*]	2.3644
PtpAffx.124621.1.S1_s_at	B9RBQ8	cytochrome P450, putative [*Ricinus communis*]	2.3608
PtpAffx.9218.1.S1_at	Q8RY17	RFO1 (RESISTANCE TO FUSARIUM OXYSPORUM 1); kinase [*Arabidopsis thaliana*]	2.3591
PtpAffx.146585.1.A1_at	Q9T0L5	calcium-binding EF hand family protein [*Arabidopsis thaliana*]	2.3552
PtpAffx.94296.1.S1_at	B9T345	carbonic anhydrase, putative [*Ricinus communis*]	2.3538
Ptp.4984.1.S1_s_at	B9HKB0	early light-inducible protein [*Populus trichocarpa*]	2.3511
Ptp.629.1.S1_at	B9T4J5	prolyl 4-hydroxylase alpha subunit, putative [*Ricinus communis*]	2.3467
PtpAffx.200557.1.S1_at	Q9SJK3	Argonaute (AGO1)-like protein [*Arabidopsis thaliana*]	2.3384
PtpAffx.218532.1.S1_s_at	O82333	auxin-responsive GH3 family protein [*Arabidopsis thaliana*]	2.3382
PtpAffx.5074.1.A1_at	Q43587	PAR-1a [*Nicotiana tabacum*]	2.3376
PtpAffx.34924.1.S1_at	B9RS14	serine-threonine protein kinase, plant-type, putative [*Ricinus communis*]	2.3347

Table S2. *Cont.*

PtpAffx.50909.1.A1_s_at	B9RZV5	RNA binding protein, putative [*Ricinus communis*]	2.3167
PtpAffx.201602.1.S1_s_at	Q9STX6	pathogenesis-related thaumatin family protein(Thaumatin-like protein) [*Arabidopsis thaliana*]	2.3142
PtpAffx.47796.1.S1_s_at	B9SXA4	Stachyose synthase precursor, putative [*Ricinus communis*]	2.3103
PtpAffx.20179.3.A1_at	B9H346	ubiquitin [*Solanum lycopersicum*]	2.3096
PtpAffx.72392.1.A1_at	B9GHU3	GH3 family protein [*Populus trichocarpa*]	2.2979
Ptp.6264.1.S1_s_at	B9TAG3	metal ion binding protein, putative [*Ricinus communis*]	2.2885
Ptp.8062.1.S1_s_at	A9PFE0	glutaredoxin [*Populus trichocarpa*]	2.2835
Ptp.732.1.A1_at	B9SB02	Reticuline oxidase precursor, putative [*Ricinus communis*]	2.2824
PtpAffx.224978.1.S1_s_at	Q9LMN8	WAK3 (wall associated kinase 3); kinase/ protein serine/threonine kinase [*Arabidopsis thaliana*]	2.2799
PtpAffx.21524.1.S1_s_at	B9RPP7	DUF26 domain-containing protein 2 precursor, putative [*Ricinus communis*]	2.2697
PtpAffx.491.4.S1_at	A9PA59	small molecular heat shock protein 17.5 [*Nelumbo nucifera*]	2.2694
PtpAffx.125908.1.A1_at	Q6WEQ2	F box protein family [*Arabidopsis lyrata* subsp. lyrata]	2.2595
PtpAffx.70153.1.A1_at	Q8L7M7	phosphoinositide binding [*Arabidopsis thaliana*]	2.2576
PtpAffx.1286.1.S1_s_at	B9SIY7	*s*-adenosylmethionine decarboxylase, putative [*Ricinus communis*]	2.2503
Ptp.5927.1.S1_s_at	B9UD35	seed imbibition protein 1 [*Vitis vinifera*]	2.2494
PtpAffx.203048.1.S1_at	O81760	protein phosphatase 2C family protein/PP2C family protein [*Arabidopsis thaliana*]	2.2475
Ptp.5638.1.S1_at	B9H5H8	cyclin B, putative [*Ricinus communis*]	2.2437
Ptp.1869.2.S1_s_at	A7Y7H5	putative S-adenosylmethionine decarboxylase [*Prunus dulcis*]	2.2429
Ptp.8014.1.S1_at	A0FJY4	NIMIN2c protein [*Nicotiana tabacum*]	2.2421
PtpAffx.158598.1.S1_s_at	Q8S950	kinesin-like protein NACK1 [*Nicotiana tabacum*]	2.2395
Ptp.4034.1.S1_at	B6U0V6	Zeamatin precursor, putative [*Ricinus communis*]	2.2358
PtpAffx.56737.2.A1_a_at	B9S1Y4	cell division cycle, putative [*Ricinus communis*	2.235
Ptp.5017.1.S1_at	B9SJ54	big map kinase/bmk, putative [*Ricinus communis*]	2.2345
PtpAffx.132628.1.S1_s_at	Q6R7N3	putative WRKY transcription factor 30 [*Vitis aestivalis*]	2.2339
Ptp.5536.1.S1_at	Q9FQE4	glutathione S-transferase GST 14 [*Glycine max*]	2.228
PtpAffx.97959.1.A1_at	B9P7I4	white-brown-complex ABC transporter family [*Populus trichocarpa*]	2.2153
PtpAffx.7896.4.A1_a_at	B9P9S8	chalcone synthase[*Populus trichocarpa*]	2.2142

Table S2. *Cont.*

PtpAffx.18145.1.A1_at	A5YVF1	Protein SUPPRESSOR OF GENE SILENCING 3	2.2134
Ptp.3600.1.S1_at	Q94G59	sinapyl alcohol dehydrogenase [*Populus tremuloides*]	2.2115
PtpAffx.203070.1.S1_s_at	Q9SD20	male sterility MS5, putative [*Arabidopsis thaliana*]	2.2054
PtpAffx.215757.1.S1_s_at	Q0WML0	ABC transporter family protein [*Arabidopsis thaliana*]	2.1987
Ptp.2894.1.A1_at	B9HRU0	cytochrome P450 [*Populus trichocarpa*]	2.1906
PtpAffx.209749.1.S1_at	Q84LB2	(E,E)-alpha-farnesene synthase. [*Malus* × *domestica*]	2.1883
PtpAffx.34370.1.S1_at	B9T8B5	transcription factor, putative [*Ricinus communis*]	2.1851
PtpAffx.46665.1.S1_s_at	B6V6Z9	GAST-like protein [*Populus euphratica*]	2.1839
Ptp.6735.1.S1_a_at	B9T4Z2	zinc finger protein, putative [*Ricinus communis*]	2.1692
PtpAffx.152207.1.S1_at	B9HJH7	rna-dependent RNA polymerase [*Populus trichocarpa*]	2.167
PtpAffx.203314.1.S1_x_at	Q9M4E8	heat shock protein 70. [*Cucumis sativus*]	2.1632
PtpAffx.210066.1.S1_at	Q42670	1-acyl-sn-glycerol-3-phosphate acyltransferase[*Cocos nucifera*]	2.1614
PtpAffx.27098.1.S1_at	B9GJN3	glycoside hydrolase [*Populus trichocarpa*]	2.1588
PtpAffx.27882.1.S1_at	Q9SW07	AtGDU2 (*Arabidopsis thaliana* GLUTAMINE DUMPER 2)	2.1574
PtpAffx.249.529.A1_at	B9SMS2	ATP binding protein, putative [*Ricinus communis*]	2.1554
PtpAffx.221435.1.S1_at	Q9ZSP5	subtilisin-like protease (AIR3) [*Arabidopsis thaliana*]	2.1519
PtpAffx.83652.1.A1_at	A8CCN3	DC1 domain containing protein [*Nicotiana tabacum*]	2.1506
PtpAffx.10247.1.A1_a_at	Q6ZXI3	putative calmodulin-like protein [*Populus* × *canadensis*]	2.1378
PtpAffx.828.1.A1_x_at	B9RBC9	structural constituent of cell wall, putative [*Ricinus communis*]	2.1375
PtpAffx.133756.2.A1_at	B9T8G8	Homeobox-leucine zipper protein HAT14, putative [*Ricinus communis*]	2.1342
PtpAffx.221169.1.S1_at	Q9FMZ4	transcriptional factor B3 family protein [*Arabidopsis thaliana*]	2.1293
PtpAffx.249.87.A1_x_at	O74346	cell agglutination protein Map4 [*Schizosaccharomyces pombe* 972h-]	2.1281
Ptp.6999.1.S1_at	B9RDG0	copper ion binding protein, putative [*Ricinus communis*]	2.1226
PtpAffx.214377.1.S1_s_at	Q6PQQ3	ovule development protein, putative (AP2-like ethylene-responsive transcription factor AIL5 (Protein AINTEGUMENTA-LIKE 5) [*Arabidopsis thaliana*]	2.1219
PtpAffx.26590.3.A1_a_at	Q93ZM9	JAZ10 (JASMONATE-ZIM-DOMAIN PROTEIN 10) [*Arabidopsis thaliana*]	2.1194
Ptp.4034.2.S1_x_at	B9SZE5	Zeamatin precursor, putative [*Ricinus communis*]	2.1182
PtpAffx.219808.1.S1_at	Q9LZR0	homeodomain protein Putative homeobox-leucine zipper protein ATHB-51 (Homeodomain transcription factor ATHB-51) (HD-ZIP protein ATHB-51) [*Arabidopsis thaliana*]	2.1169

Table S2. *Cont.*

PtpAffx.75868.1.A1_at	Q9SJW9	CDT1A (ARABIDOPSIS HOMOLOG OF YEAST CDT1 A); cyclin-dependent protein kinase/ protein binding [*Arabidopsis thaliana*]	2.1067
PtpAffx.50058.1.A1_a_at	Q9LTZ5	zinc finger (C3HC4-type RING finger) family protein [*Arabidopsis thaliana*]	2.1065
PtpAffx.203419.1.S1_at	Q84WJ2	Probable DNA primase large subunit	2.1043
PtpAffx.135961.2.A1_s_at	Q60D04	Myb-like DNA-binding protein, putative [*Solanum demissum*]	2.0854
PtpAffx.152367.1.S1_s_at	B9RNW4	WRKY transcription factor, putative [*Ricinus communis*]	2.0796
PtpAffx.163418.2.S1_s_at	B9SPH2	protein binding protein, putative [*Ricinus communis*]zinc finger (C3HC4-type RING finger) family	2.0748
PtpAffx.17241.1.S1_at	B9R8T6	cinnamoyl-CoA reductase, putative [*Ricinus communis*]	2.0707
PtpAffx.211300.1.S1_at	Q38944	Probable steroid reductase DET2 [*Arabidopsis thaliana*]	2.0705
PtpAffx.93558.1.A1_at	B9R8I4	Anthranilate N-benzoyltransferase protein, putative [*Ricinus communis*]	2.0688
PtpAffx.34524.3.A1_a_at	B9H6Q2	AP2 domain-containing transcription factor [*Populus trichocarpa*]	2.0643
PtpAffx.204882.1.S1_at	Q1PDX3	[*Arabidopsis thaliana*]	2.0634
PtpAffx.147445.1.A1_at	Q9LNK3	leucine-rich repeat family protein/protein kinase family protein [*Arabidopsis thaliana*]	2.0602
Ptp.1111.1.A1_s_at	B9S6C1	Benzoate carboxyl methyltransferase, putative [*Ricinus communis*]	2.0595
Ptp.7280.1.S1_at	B9SNC0	serine-threonine protein kinase, plant-type, putative [*Ricinus communis*]	2.058
PtpAffx.46220.1.S1_at	B6U8T2	growth-regulating factor 1 [*Zea mays*]	2.0534
PtpAffx.224930.1.S1_at	O48723	PLA2A (PHOSPHOLIPASE A 2A); lipase/ nutrient reservoir [*Arabidopsis thaliana*]	2.0481
PtpAffx.213578.1.S1_s_at	Q9FFJ5	zinc finger (C3HC4-type RING finger) protein family [*Arabidopsis thaliana*]	2.0447
PtpAffx.200701.1.S1_s_at	Q9FMR3	No apical meristem (NAM) protein family [*Arabidopsis thaliana*]	2.0432
PtpAffx.214589.1.S1_at	Q9SII6	protein kinase, putative [*Arabidopsis thaliana*]	2.0422
PtpAffx.20179.2.S1_at	Q40164	ubiquitin [*Solanum lycopersicum*]	2.0378
PtpAffx.7903.2.A1_s_at	B9SLW2	ctp synthase, putative [*Ricinus communis*]	2.0373
PtpAffx.148582.1.S1_at	B9RIC0	origin recognition complex subunit, putative [*Ricinus communis*]	2.0294
Ptp.288.1.A1_s_at	Q03682	Luminal-binding protein 2; Short=BiP 2;	2.0284
Ptp.21.1.S1_s_at	B9HQL3	histone deacetylase [*Populus trichocarpa*]	2.0258
PtpAffx.200385.1.S1_at	Q9SUQ2	Ethylene-responsive transcription factor CRF2	2.0245
Ptp.114.1.S1_at	Q9FVF7	putative AUX1-like permease [*Populus tremula* × *Populus tremuloides*]	2.0194
PtpAffx.2625.3.A1_a_at	B9SUZ4	alcohol dehydrogenase, putative [*Ricinus communis*]	2.0155

Table S2. *Cont.*

PtpAffx.77821.1.A1_at	B9R8A6	Zeamatin precursor, putative [*Ricinus communis*]	2.0145
Ptp.1433.1.S1_at	B9T1Q8	heat shock protein, putative [*Ricinus communis*]	2.0117
PtpAffx.1802.2.S1_at	B9MVS6	Heat shock transcription factor (HSF) [*Glycine max*]	2.0112
PtpAffx.211298.1.S1_s_at	P42813	ribonuclease 1 (RNS1) [*Arabidopsis thaliana*]	2.0106
Ptp.4093.1.S1_at	Q6E593	benzoyl coenzyme A: benzyl alcohol benzoyl transferase [*Petunia × hybrida*]	2.0104
PtpAffx.214491.1.S1_at	O80786	minichromosome maintenance family protein/MCM family protein [*Arabidopsis thaliana*]	2.0096
PtpAffx.100788.1.S1_a_at	B9T578	ARF GTPase activator, putative [*Ricinus communis*]	2.0065
PtpAffx.58533.1.S1_at	B9HTX8	beta-ketoacyl-coa synthase family protein [*Populus trichocarpa*]	2.0027
Ptp.7320.1.S1_a_at	B9RSH8	ubiquitin, putative [*Ricinus communis*]	2.0015

Table S3. Down-regulated genes in Affymetrix microarray analysis.

**Probe Set ID**	**Protein Acc No.**	**Description**	**Fold change (*dwf1*/WT)**
PtpAffx.216199.1.S1_at	Q94A08	AtSIP2 (*Arabidopsis thaliana* seed imbibition 2); hydrolase, hydrolyzing O-glycosyl compounds[*Arabidopsis thaliana*]	0.499
PtpAffx.213177.1.S1_at	Q9LHJ2	UDP-glucose glucosyltransferase-like protein [*Arabidopsis thaliana*]	0.4987
Ptp.7536.1.A1_at	P93788	REMO_SOLTU Remorin [*Solanum tuberosum*]	0.4986
PtpAffx.16539.1.A1_s_at	Q39436	SIEP1L protein [*Beta vulgaris* subsp. vulgaris]	0.4978
PtpAffx.52630.1.A1_at	B9H902	aquaporin, MIP family, TIP subfamily [*Populus trichocarpa*]	0.4976
PtpAffx.342.1.S1_s_at	B9T061	acyltransferase, putative [*Ricinus communis*]	0.4968
PtpAffx.68529.1.S1_at	B9IBX4	SAUR family protein [*Populus trichocarpa*]	0.4965
Ptp.3303.1.S1_at	B9SDI4	bel1 homeotic protein, putative [*Ricinus communis*]	0.4965
PtpAffx.117004.2.S1_at	B9S1P7	pectin acetylesterase, putative [*Ricinus communis*]	0.4957
PtpAffx.29666.2.S1_at	B6TKI4	MAPK activating protein [*Zea mays*]	0.4946
PtpAffx.68024.1.S1_at	Q9FVQ4	membrane protein, putative [*Arabidopsis thaliana*]	0.4938
PtpAffx.11959.1.A1_at	Q9SSG2	nodulin family protein [*Arabidopsis thaliana*]	0.4935

Table S3. *Cont.*

PtpAffx.214389.1.S1_at	Q9LUM3	Subtilisin proteinase-like protein [*Arabidopsis thaliana*]	0.4934
PtpAffx.147430.1.A1_at	B9I4F3	AP2/ERF domain-containing transcription factor [*Populus trichocarpa*]	0.4931
Ptp.128.1.S1_at	Q8RW15	aux/IAA protein [Populus tremula × *Populus tremuloides*]	0.4928
PtpAffx.92412.1.S1_at	B9SI27	Epidermis-specific secreted glycoprotein EP1 precursor, putative [*Ricinus communis*]	0.4926
PtpAffx.204253.1.S1_at	Q56YQ6	kinesin motor family protein[*Arabidopsis thaliana*]	0.4911
PtpAffx.207364.1.S1_at	Q93VJ4	basic helix-loop-helix (bHLH) family protein [*Arabidopsis thaliana*]	0.4903
PtpAffx.101087.1.S1_a_at	O49675	NCED4 (NINE-CIS-EPOXYCAROTENOID DIOXYGENASE 4) [*Arabidopsis thaliana*]	0.4902
PtpAffx.7100.1.S1_at	B9RYN8	Protein ABIL2, putative [*Ricinus communis*]	0.49
PtpAffx.224677.1.S1_at	Q8LPI3	MATE efflux family protein [*Arabidopsis thaliana*]	0.4898
PtpAffx.209542.1.S1_at	Q9LZS7	GDSL-motif lipase/hydrolase protein [*Arabidopsis thaliana*]	0.4894
PtpAffx.28083.1.S1_at	B9RXC7	coproporphyrinogen III oxidase, putative [*Ricinus communis*]	0.4891
PtpAffx.16288.1.S1_at	Q9LUZ6	PMR5 (POWDERY MILDEW RESISTANT 5) [*Arabidopsis thaliana*]	0.4888
PtpAffx.123395.1.S1_at	B9N2J5	auxin-induced protein [*Populus trichocarpa*]	0.4888
PtpAffx.211115.1.S1_at	Q9SS64	MATE efflux family protein [*Arabidopsis thaliana*]	0.4887
PtpAffx.128519.1.S1_at	B9S8H0	zinc finger protein, putative [*Ricinus communis*]	0.4886
PtpAffx.43798.1.A1_a_at	B6TWK6	fiber expressed protein [*Zea mays*]	0.4886
PtpAffx.2814.1.A1_at	B9SFE5	ubiquitin-protein ligase, putative [*Ricinus communis*]	0.4885
PtpAffx.1910.1.S1_at	B9RSH9	Pectinesterase PPE8B precursor, putative [*Ricinus communis*]	0.4884
PtpAffx.203255.1.S1_s_at	Q9CAD6	cytochrome P450, putative [*Arabidopsis thaliana*]	0.4878
PtpAffx.20744.1.A1_at	B9RTU6	basic 7S globulin 2 precursor small subunit, putative [*Ricinus communis*]	0.4878
PtpAffx.220980.1.S1_s_at	O48925	cytochrome P450 82C1 - soybean. [*Glycine max*]	0.4874
PtpAffx.5070.1.A1_at	B9S6F6	abhydrolase domain containing, putative [*Ricinus communis*]	0.4867
PtpAffx.38333.1.S1_at	B9RXM4	multidrug resistance pump, putative [*Ricinus communis*]	0.4861
PtpAffx.118623.2.S1_at	B9SP81	phosphoglucomutase, putative [*Ricinus communis*]	0.4859
PtpAffx.208531.1.S1_at	Q42910	Pyruvate, phosphate dikinase, chloroplast precursor (Pyruvate, orthophosphate dikinase) [*Mesembryanthemum crystallinum*]	0.4851
PtpAffx.19134.1.A1_a_at	Q8H9B7	putative lipid transfer protein [*Solanum tuberosum*]	0.4837
PtpAffx.7804.3.A1_a_at	B9RS62	Dehydration-responsive protein RD22 precursor, putative [*Ricinus communis*]	0.4824

Table S3. *Cont.*

Ptp.962.1.S1_s_at	Q2LAK1	cytochrome P450 monooxygenase CYP77A3v2 [*Glycine max*]	0.4822
PtpAffx.14429.1.A1_a_at	B9RXD8	short-chain dehydrogenase, putative [*Ricinus communis*]	0.482
PtpAffx.160443.1.A1_s_at	B9HZ04	calmodulin-binding protein [*Arabidopsis thaliana*]	0.4818
PtpAffx.12219.1.S1_s_at	B9SSJ0	zinc finger protein, putative [*Ricinus communis*]	0.4816
Ptp.7552.1.S1_s_at	A8Y5V3	alpha galactosidase precursor [*Coffea arabica*]	0.4814
PtpAffx.1251.1.S1_at	B9IAJ4	nitrate transporter, H+/oligopeptide symporter POT family [*Populus trichocarpa*]	0.4814
PtpAffx.101550.1.A1_at	B9IF32	methyltransferase [*Arabidopsis thaliana*]	0.4805
PtpAffx.220064.1.S1_at	Q8LPK2	multidrug resistance P-glycoprotein, putative (ABC transporter B family member 2) [*Arabidopsis thaliana*]	0.4804
PtpAffx.200885.1.S1_x_at	Q6NLB1	F-box family protein [*Arabidopsis thaliana*]	0.4794
PtpAffx.209715.1.S1_at	Q9SCV4	BGAL8 (beta-galactosidase 8) ; beta-galactosidase/ catalytic/ cation binding/sugar binding [*Arabidopsis thaliana*]	0.479
PtpAffx.13217.1.S1_at	A0ZNJ9	pectin methylesterase 1 [*Pyrus communis*]	0.4782
PtpAffx.207964.1.S1_at	Q4V3D9	strictosidine synthase family protein [*Arabidopsis thaliana*]	0.4776
Ptp.1460.1.S1_at	Q20BM9	UPF0497 membrane protein[*Arabidopsis thaliana*]	0.4774
PtpAffx.118623.1.S1_at	D1I0G7	similar to phosphoglucomutase, putative/glucose phosphomutase, putative [*Vitis vinifera*]	0.4766
PtpAffx.200885.1.S1_at	Q8RY82	F-box family protein [*Arabidopsis thaliana*]	0.4765
PtpAffx.54813.1.A1_at	Q9XEF0	basic helix-loop-helix family protein [*Arabidopsis thaliana*]	0.4754
PtpAffx.26424.2.S1_a_at	B9SLF3	Gibberellin-regulated protein 1 precursor, putative [*Ricinus communis*]	0.475
Ptp.2631.1.S1_at	B9R9C1	Remorin, putative [*Ricinus communis*]	0.4733
PtpAffx.219012.1.S1_s_at	Q9LYQ7	transferase family protein [*Arabidopsis thaliana*]	0.4729
PtpAffx.113258.1.S1_at	B9T6J8	ATP binding protein, putative [*Ricinus communis*]	0.4698
PtpAffx.158492.1.S1_at	B9SL40	asparagine synthetase, putative [*Ricinus communis*]	0.4696
PtpAffx.36812.1.A1_a_at	B3V945	PAE pectinacetylesterase family protein [*Litchi chinensis*]	0.4694
PtpAffx.47488.1.S1_s_at	B9S732	Patellin-3, putative [*Ricinus communis*]	0.4693
PtpAffx.23593.3.A1_at	B9SVU8	UDP-glucuronosyltransferase, putative [*Ricinus communis*]	0.469
Ptp.5207.1.S1_at	B9RSI5	spike-1, putative [*Ricinus communis*]Guanine nucleotide exchange factor	0.4687
PtpAffx.210907.1.S1_at	Q1W7B0	Chloroplast heat shock protein-binding protein [*Arabidopsis thaliana*]	0.4683
PtpAffx.15891.1.A1_at	B9S7Q3	zinc finger protein, putative [*Ricinus communis*]	0.4669

Table S3. *Cont.*

PtpAffx.114066.1.A1_at	B9SG70	Hippocampus abundant transcript 1 protein, putative [*Ricinus communis*]	0.4663
PtpAffx.29385.1.A1_at	B9S5Y4	Monoglyceride lipase, putative [*Ricinus communis*]	0.4662
PtpAffx.213339.1.S1_at	Q8RWI9	ABC transporter family protein [*Arabidopsis thaliana*]	0.4658
PtpAffx.218352.1.S1_at	Q8VZ70	protein kinase family [*Arabidopsis thaliana*]	0.4643
PtpAffx.86833.1.A1_at	Q43614	DNA-binding protein [*Petunia x hybrida*]	0.4642
PtpAffx.44969.1.S1_a_at	B9RSV8	pyruvate, phosphate dikinase, chloroplast, putative [*Ricinus communis*]	0.4639
PtpAffx.158231.1.A1_at	B6EWY1	UDP-glucose:glucosyltransferase [*Lycium barbarum*]	0.4638
PtpAffx.208033.1.S1_at	Q9C9F4	zinc finger (B-box type) family protein [*Arabidopsis thaliana*]	0.4619
PtpAffx.95760.1.A1_a_at	B9GET3	beta-ketoacyl-coa synthase family protein [*Populus trichocarpa*]	0.4614
Ptp.2657.1.S1_s_at	B9RK64	beta-galactosidase, putative [*Ricinus communis*]	0.46
PtpAffx.224655.1.S1_at	Q9XF43	very-long-chain fatty acid condensing enzyme (CUT1) [*Arabidopsis thaliana*]	0.4592
PtpAffx.18176.1.A1_at	B9S6L0	lipid binding protein, putative [*Ricinus communis*]	0.4589
PtpAffx.154411.1.S1_s_at	B9S985	triacylglycerol lipase, putative [*Ricinus communis*]	0.4588
PtpAffx.213622.1.S1_at	Q9M022	zinc finger (C3HC4-type RING finger) protein family [*Arabidopsis thaliana*]	0.4584
PtpAffx.204880.1.S1_at	Q9C8J6	lipase class 3 family protein [*Arabidopsis thaliana*]	0.4581
Ptp.4360.1.S1_s_at	B9RXK1	lactoylglutathione lyase, putative [*Ricinus communis*]	0.4576
PtpAffx.209595.1.S1_at	O65399	Putative glucan endo-1,3-beta-glucosidase 1 precursor (1-3) -beta-glucan endohydrolase) (1-3-beta-glucanase) (Beta-1,3-endoglucanase) (Beta-1,3-glucanase) [*Arabidopsis thaliana*]	0.4574
PtpAffx.790.1.A1_at	B9IQS4	fasciclin-like arabinogalactan protein 9.2 [*Populus trichocarpa*]	0.4573
PtpAffx.150066.1.S1_at	B9RII2	ATP binding protein, putative [*Ricinus communis*]	0.4569
Ptp.2379.2.S1_a_at	B9RB49	aldehyde dehydrogenase, putative [*Ricinus communis*]	0.4554
Ptp.2234.1.S1_s_at	B9IDR8	similar to pectin methylesterase 1 [*Vitis vinifera*]	0.4553
PtpAffx.84732.1.S1_s_at	A9XNQ3	protease inhibitor/seed storage/lipid transfer protein (LTP)	0.4541
PtpAffx.87526.1.S1_at	B9N551	ATP binding protein, putative [*Ricinus communis*]	0.454
PtpAffx.77416.1.A1_at	B9P9D5	predicted protein	0.4536
PtpAffx.200077.1.S1_s_at	Q94AN4	ovule development protein, putative(AP2-like ethylene-responsive transcription factor At1g16060) [*Arabidopsis thaliana*]	0.4527

Table S3. *Cont.*

PtpAffx.136997.1.S1_s_at	Q3E882	zinc finger (C3HC4-type RING finger) family protein [*Arabidopsis thaliana*]	0.4527
PtpAffx.16221.1.A1_a_at	O81373	cotton fiber expressed protein 1 [*Gossypium hirsutum*]	0.4518
PtpAffx.209025.1.S1_at	O48786	putative cytochrome P450 [*Arabidopsis thaliana*]	0.4497
PtpAffx.5092.2.S1_at	B9HYU1	anthocyanidin reductase [*Populus trichocarpa*]	0.4497
Ptp.7912.1.S1_at	B9ILW8	autoinhibited H+ ATPase [*Populus trichocarpa*]	0.4489
PtpAffx.68429.2.S1_at	B9S8C8	WRKY transcription factor, putative [*Ricinus communis*]	0.4489
PtpAffx.4462.1.S1_at	A2TM13	beta-1,3-glucanase [*Hevea brasiliensis*]	0.4488
PtpAffx.222760.1.S1_x_at	P09789	Glycine-rich cell wall structural protein 1 precursor [*Petunia* × *hybrida*]	0.448
PtpAffx.22300.1.S1_at	B9RKG5	dipeptidase, putative [*Ricinus communis*]	0.447
PtpAffx.13272.2.S1_a_at	Q6T1C8	peroxidase precursor [*Quercus suber*]	0.4437
Ptp.5997.1.S1_at	B9IFG0	hydroxycinnamoyl CoA shikimate/quinate hydroxycinnamoyltransferase-like protein [*Populus trichocarpa*]	0.4433
PtpAffx.60700.1.S1_at	Q94C21	eukaryotic translation initiation factor-related [*Arabidopsis thaliana*]	0.4426
PtpAffx.220056.1.S1_x_at	P10496	Glycine-rich cell wall structural protein 1.8 precursor (GRP 1.8) [*Phaseolus vulgaris*]	0.4415
PtpAffx.152938.1.A1_at	B9T3V0	phosphoprotein phosphatase, putative [*Ricinus communis*]	0.4408
Ptp.3476.1.S1_s_at	A9PHG1	ubiquitin [*Pisum sativum*]	0.4401
PtpAffx.22909.1.A1_at	Q9ZVS5	extracellular dermal glycoprotein, putative/EDGP, putative [*Arabidopsis thaliana*]	0.4395
PtpAffx.20635.1.A1_at	B9RYN0	3-oxoacyl-[acyl-carrier-protein] synthase, putative [*Ricinus communis*]	0.439
PtpAffx.121543.1.A1_at	B9S1S9	9-cis-epoxycarotenoid dioxygenase, putative [*Ricinus communis*]	0.4383
PtpAffx.139063.1.S1_at	A7MAS5	UDP-glucose:flavonoid 7-*O*-glucosyltransferase [*Malus x domestica*]	0.4381
PtpAffx.23593.1.S1_at	B9SVU7	UDP-glucuronosyltransferase, putative [*Ricinus communis*]	0.438
PtpAffx.42125.2.A1_at	B9S983	triacylglycerol lipase, putative [*Ricinus communis*]	0.4377
PtpAffx.13485.1.A1_at	B9S600	diphosphonucleoside phosphohydrolase, putative [*Ricinus communis*]	0.4375
PtpAffx.127102.1.A1_at	B9T3Z8	nutrient reservoir, putative [*Ricinus communis*]	0.4358
PtpAffx.16052.1.S1_a_at	B9RXE2	Alternative oxidase 4, chloroplast precursor, putative [*Ricinus communis*]	0.435
PtpAffx.204268.1.S1_at	P33081	Auxin-induced protein 15A[*Glycine max* (soybean)]	0.4348
PtpAffx.88104.1.A1_at	B9MZY6	glutaredoxin [*Populus trichocarpa*]	0.4346

Table S3. *Cont.*

PtpAffx.2103.1.A1_s_at	B9HPN2	predicted protein [*Populus trichocarpa*]	0.4336
PtpAffx.218091.1.S1_at	O04083	hydrolase, alpha/beta fold family protein [*Arabidopsis thaliana*]	0.4328
PtpAffx.134656.1.A1_at	B9RT47	RALFL33, putative [*Ricinus communis*]	0.4324
Ptp.4947.1.S1_s_at	B9MZ14	predicted protein [*Populus trichocarpa*]	0.4311
PtpAffx.15925.1.S1_at	B9H736	predicted protein [*Populus trichocarpa*]	0.431
PtpAffx.20007.1.A1_at	B9RRI4	mitochondrial carrier protein, putative [*Ricinus communis*]	0.4302
PtpAffx.133884.1.A1_at	B9I1M81	predicted protein [*Populus trichocarpa*]	0.43
PtpAffx.132702.1.A1_s_at	B9RA86	alpha-2,8-sialyltransferase 8b, putative [*Ricinus communis*]	0.4295
PtpAffx.113991.1.A1_at	B2LWX9	E2 ubiquitin-conjugating enzyme UBC10 [*Brassica napus*]	0.4293
PtpAffx.976.1.A1_at	B9SC75	triacylglycerol lipase, putative [*Ricinus communis*]	0.4285
PtpAffx.14721.1.S1_at	B9HAI5	predicted protein [*Populus trichocarpa*]	0.4281
PtpAffx.120153.1.S1_s_at	A2TEI7	xyloglucan endotransglycosylase/hydrolase precursor XTH-36 [*Populus tremula* × *Populus tremuloides*]	0.4249
PtpAffx.17584.1.A1_at	Q0ZIH9	UP-9A [*Nicotiana tabacum*]	0.4245
PtpAffx.4350.1.A1_s_at	Q5PYQ5	chloroplast oxygen-evolving enhancer protein [*Manihot esculenta*]	0.4244
PtpAffx.8616.1.A1_at	C0Z2J1	universal stress protein (USP) family protein [*Arabidopsis thaliana*]	0.4238
PtpAffx.58121.1.S1_at	Q0VH88	3-ketoacyl-CoA reductase 1 [*Gossypium hirsutum*]	0.4237
PtpAffx.211760.1.S1_s_at	Q9LZI3	invertase/pectin methylesterase inhibitor family protein [*Arabidopsis thaliana*]	0.4234
PtpAffx.132310.1.A1_at	B9S4X3	UDP-glucosyltransferase, putative [*Ricinus communis*]	0.4224
PtpAffx.99814.1.A1_at	B9HZ69	predicted protein [*Populus trichocarpa*]	0.4218
PtpAffx.113871.1.A1_at	B9I586	predicted protein [*Populus trichocarpa*]	0.4204
PtpAffx.126959.1.A1_at	B9H4S9	predicted protein [*Populus trichocarpa*]	0.42
PtpAffx.201584.1.S1_s_at	Q8S901	predicted protein [*Populus trichocarpa*]	0.4189
PtpAffx.214390.1.S1_at	Q8L8U9	integral membrane family protein [*Arabidopsis thaliana*]	0.4188
PtpAffx.26469.3.A1_a_at	B9S9A5	catalytic, putative [*Ricinus communis*]	0.4176
PtpAffx.39778.1.A1_at	Q8RW16	aux/IAA protein [Populus tremula × *Populus tremuloides*]	0.4174
PtpAffx.203043.1.S1_at	Q9LYZ6	transferase family [*Arabidopsis thaliana*]	0.4166
PtpAffx.24378.1.S1_at	B6TQ19	lysM domain containing protein [Zea mays]	0.4152
PtpAffx.27718.1.S1_s_at	Q93V70	zinc-binding family protein [*Arabidopsis thaliana*]	0.4145

Table S3. *Cont.*

Ptp.7026.1.S1_s_at	B9ICH8	predicted protein [*Populus trichocarpa*]	0.4142
PtpAffx.205671.1.S1_at	Q67ZI9	GDSL-motif lipase/hydrolase family protein [*Arabidopsis thaliana*]	0.4121
PtpAffx.67394.1.S1_at	B9RP24	14 kDa proline-rich protein DC2.15 precursor, putative [*Ricinus communis*]	0.4112
Ptp.5472.1.S1_at	B9HNJ2	tubulin, beta chain [*Populus trichocarpa*]	0.411
PtpAffx.209713.1.S1_at	Q9SXA6	Bifunctional nuclease bfn1 (Putative bifunctional nuclease bfn1)	0.4083
Ptp.171.1.A1_at	B9IBS9	glutaredoxin [*Populus trichocarpa*]	0.4068
PtpAffx.122358.1.S1_s_at	B9RC10	glucose-methanol-choline (gmc) oxidoreductase, putative [*Ricinus communis*]	0.4064
PtpAffx.21413.1.S1_at	B9RPB1	sigma factor sigb regulation protein rsbq, putative [*Ricinus communis*]	0.4056
PtpAffx.152696.1.S1_at	B9R7B0	triacylglycerol lipase, putative [*Ricinus communis*]	0.4044
PtpAffx.133294.1.A1_at	A9PIN7	predicted protein [*Populus trichocarpa*]	0.4033
PtpAffx.146651.1.A1_at	B9I1Y0	predicted protein [*Populus trichocarpa*]	0.4032
Ptp.127.1.S1_s_at	Q8RW14	aux/IAA protein [Populus tremula × *Populus tremuloides*]	0.4027
PtpAffx.18446.1.A1_at	B9RP35	ATP binding protein, putative [*Ricinus communis*]	0.3956
PtpAffx.139572.1.S1_s_at	Q8GT67	xyloglucan-specific fungal endoglucanase inhibitor protein precursor	0.3942
PtpAffx.201596.1.S1_at	Q9C7F8	multidrug resistance *P*-glycoprotein, putative (ABC transporter B family member 13) [*Arabidopsis thaliana*]	0.3913
PtpAffx.209702.1.S1_at	Q0WQA8	disease resistance protein (TIR class), putative [*Arabidopsis thaliana*]	0.3898
PtpAffx.224739.1.S1_at	Q9FK76	subtilisin-related protease [*Arabidopsis thaliana*]	0.389
PtpAffx.45694.1.S1_at	B9GG65	f-box family protein [*Populus trichocarpa*]	0.3875
PtpAffx.5230.1.A1_at	B9HYX6	predicted protein [*Populus trichocarpa*]	0.3862
PtpAffx.219331.1.S1_s_at	Q9C6G5	leucine-rich repeat family protein/protein kinase family protein [*Arabidopsis thaliana*]	0.386
Ptp.6356.1.S1_s_at	B9T394	endomembrane-associated protein, putative [*Ricinus communis*]	0.3849
PtpAffx.64132.1.S1_at	Q07423.1	Hexose carrier protein HEX6[*Arabidopsis thaliana*]	0.3846
PtpAffx.200461.1.S1_at	Q66GI5	glucose-methanol-choline (GMC) oxidoreductase family protein [*Arabidopsis thaliana*]	0.3822
PtpAffx.205809.1.S1_at	Q9FH56	leucine rich repeat protein family [*Arabidopsis thaliana*]	0.3808
PtpAffx.50721.1.A1_s_at	B9S1N3	receptor serine-threonine protein kinase, putative [*Ricinus communis*]	0.3798
PtpAffx.107986.1.A1_at	Q3KU27	nectarin IV [Nicotiana langsdorffii × Nicotiana sanderae]	0.3788

Table S3. *Cont.*

PtpAffx.209619.1.S1_at	Q9XEA0	leucyl-tRNA synthetase, putative/leucine--tRNA ligase, putative [*Arabidopsis thaliana*]	0.3766
Ptp.2274.1.S1_s_at	Q93W91	universal stress protein (USP) family protein [*Arabidopsis thaliana*]	0.3754
PtpAffx.209840.1.S1_s_at	Q93Z25	pectate lyase family protein [*Arabidopsis thaliana*]	0.3754
PtpAffx.27096.1.A1_at	B9N4I8	predicted protein [*Populus trichocarpa*]	0.3743
PtpAffx.12242.1.A1_at	B9S7Q1	zinc finger protein, putative [*Ricinus communis*]	0.3733
PtpAffx.24378.1.S1_a_at	Q8H053	Putative dihydrodipicolinate reductase-like protein [*Oryza sativa* (japonica cultivar-group)]	0.3715
PtpAffx.6080.1.S1_s_at	B9S606	Nuclease PA3, putative [*Ricinus communis*]	0.3709
PtpAffx.618.2.S1_a_at	Q6PML6	metallothionein 1a [*Populus trichocarpa* × *Populus deltoids*]	0.3675
PtpAffx.28262.1.S1_at	Q8VZW5	Putative uncharacterized protein [*Arabidopsis thaliana*]	0.3672
PtpAffx.214179.1.S1_at	Q9C936	2-oxoglutarate-dependent dioxygenase, putative [*Arabidopsis thaliana*]	0.3667
PtpAffx.104760.1.S1_at	B9S587	zinc finger protein, putative [*Ricinus communis*]	0.3654
PtpAffx.24363.1.S1_at	B9HPR3	predicted protein [*Populus trichocarpa*]	0.3622
PtpAffx.1770.5.S1_at	Q9M6B7	lipid transfer protein precursor [*Gossypium hirsutum*]	0.3621
PtpAffx.221374.1.S1_s_at	Q9ZWJ3	UDP-glucoronosyl/UDP-glucosyl transferase family protein [*Arabidopsis thaliana*]	0.3613
PtpAffx.211930.1.S1_at	Q9SU13	fasciclin-like arabinogalactan-protein (FLA2) [*Arabidopsis thaliana*]	0.3607
PtpAffx.54705.1.S1_at	B9H354	predicted protein [*Populus trichocarpa*]	0.3582
PtpAffx.6089.1.S1_at	A9PH23	predicted protein [*Populus trichocarpa*]	0.3581
PtpAffx.148761.1.S1_at	Q9XEA1	early-responsive to dehydration protein-related/ERD protein-related [*Arabidopsis thaliana*]	0.3577
PtpAffx.222767.1.S1_at	O04036	early-responsive to dehydration stress protein (ERD6)/sugar transporter family protein [*Arabidopsis thaliana*]	0.3548
PtpAffx.224872.1.S1_s_at	O64631	cytochrome P450, putative [*Arabidopsis thaliana*]	0.3524
Ptp.1017.1.A1_at	B9T016	sigma factor sigb regulation protein rsbq, putative [*Ricinus communis*]	0.3524
PtpAffx.75597.1.A1_at	B9HL35	predicted protein [*Populus trichocarpa*]	0.3491
PtpAffx.204357.1.S1_at	Q93ZB9	Fatty acyl-CoA reductase 3[*Arabidopsis thaliana*]	0.3481
Ptp.4050.17.A1_at	Q6PML1	metallothionein 3b [*Populus trichocarpa* × *Populus deltoides*]	0.3481

Table S3. *Cont.*

PtpAffx.101017.1.A1_at	B9RXW4	1,8-cineole synthase, chloroplast precursor, putative [*Ricinus communis*]	0.346
Ptp.5212.1.A1_at	Q00M73	membrane protein-like protein [*Glycine max*]	0.3428
PtpAffx.46298.1.S1_at	B9HK32	outward rectifying potassium channel [*Populus trichocarpa*]	0.3425
PtpAffx.19675.2.S1_s_at	B9ILL5	predicted protein [*Populus trichocarpa*]	0.342
PtpAffx.454.10.A1_at	B6UAJ2	EF hand family protein [*Zea mays*]	0.3404
PtpAffx.222905.1.S1_s_at	C0LGE0	Leucine-rich repeat receptor-like protein kinase[*Arabidopsis thaliana*]	0.3393
PtpAffx.112794.1.A1_at	C0SUC6	progesterone 5beta reductase-B [*Nicotiana tabacum*]	0.3376
PtpAffx.40421.1.A1_s_at	Q948L3	drought inducible 22 kD protein [Saccharum officinarum]	0.337
PtpAffx.74156.1.A1_at	C5I6F7	(E)-beta-caryophyllene synthase [Arabidopsis lyrata subsp. petraea]	0.3355
Ptp.3892.1.S1_at	Q9LS54	VQ motif-containing protein [*Arabidopsis thaliana*]	0.3334
Ptp.4951.1.S1_s_at	B9HNL6	DNA binding protein, putative [*Ricinus communis*]	0.3325
Ptp.5092.1.S1_at	B9MTV8	Beta-amylase, putative [*Ricinus communis*]	0.3286
PtpAffx.48123.2.A1_at	B9N558	Calcium-binding EF-hand ccd1, putative [*Ricinus communis*]	0.3275
PtpAffx.146348.1.A1_at	B9GN71	predicted protein [*Populus trichocarpa*]	0.3268
Ptp.5443.1.S1_s_at	B9RXE7	phosphofructokinase, putative [*Ricinus communis*]	0.3259
PtpAffx.59809.1.S1_at	Q8S2W4	arabinogalactan-peptide AGP23[*Arabidopsis thaliana*]	0.3249
PtpAffx.223794.1.S1_at	Q8L5C7	UDP-glucuronosyltransferase. [*Pisum sativum*]	0.3221
Ptp.953.1.A1_x_at	C5H0C1	galactinol synthase 1 [*Populus trichocarpa* × *Populus deltoides*]	0.3209
PtpAffx.2752.1.S1_at	B9GM27	annexin [*Cicer arietinum*]	0.3196
PtpAffx.11639.1.A1_at	B9H1M9	germin-like protein GLP [*Manihot esculenta*]	0.3169
Ptp.6187.1.S1_x_at	A9PG02	Bet v I allergen family protein[*Arabidopsis thaliana*]	0.3138
Ptp.6929.2.S1_a_at	B9HZA6	chaperonin-like protein [*Fischerella muscicola* PCC 7414]	0.3116
PtpAffx.147453.1.A1_at	B9I1A5	Pectate lyase precursor, putative [*Ricinus communis*]	0.3101
PtpAffx.135343.1.S1_at	B9RM18	Anthranilate N-benzoyltransferase protein, putative [*Ricinus communis*]	0.3025
PtpAffx.203137.1.S1_at	Q9SY91	protein kinase, putative [*Arabidopsis thaliana*]	0.2994
PtpAffx.108432.1.A1_at	B9GII2	Ca2+ antiporter/cation exchanger[*Populus trichocarpa*]	0.2956
PtpAffx.111903.2.S1_at	B9H841	predicted protein [*Populus trichocarpa*]	0.2945
PtpAffx.209233.1.S1_at	C0JCX4	transferase family protein [*Arabidopsis thaliana*]	0.2934
PtpAffx.200750.1.S1_at	A6H5A5	proton-dependent oligopeptide transport (POT) family protein [*Arabidopsis thaliana*]	0.2933

Table S3. *Cont.*

Ptp.6115.1.S1_at	B9I8F2	predicted protein [*Populus trichocarpa*]	0.2911
Ptp.1489.2.S1_s_at	B2BED2	Asr2 [Solanum chilense]	0.2907
PtpAffx.2130.1.A1_at	B9HHV8	laccase 90b [*Populus trichocarpa*]	0.2871
PtpAffx.105254.1.S1_at	Q8HQ02	ATP synthase F0 subunit 6 [*Thrips imaginis*]	0.2799
Ptp.4825.1.S1_at	B9RJA9	calmodulin, putative [*Ricinus communis*]	0.2794
PtpAffx.19154.1.S1_at	A9PGX3	Peroxidase 64 precursor, putative [*Ricinus communis*]	0.2791
PtpAffx.209038.1.S1_s_at	Q9FGY1	glycosyl hydrolase family 3 [*Arabidopsis thaliana*]	0.267
PtpAffx.115584.1.S1_at	B9H511	zinc finger protein, putative [*Ricinus communis*]	0.264
PtpAffx.249.35.S1_s_at	B9ILV5	zinc finger protein, putative [*Ricinus communis*]	0.2614
PtpAffx.69357.1.A1_at	B9HYQ1	ccd1, putative [*Ricinus communis*]	0.2604
PtpAffx.154539.1.S1_s_at	B9IIU8	predicted protein [*Populus trichocarpa*]	0.26
PtpAffx.158051.1.A1_at	B9GJA3	cytochrome P450 [*Populus trichocarpa*]	0.2578
PtpAffx.204285.1.S1_s_at	B9DHX5	proline-rich family protein (PRP4) [*Arabidopsis thaliana*]	0.2533
PtpAffx.4286.8.S1_at	B9N408	stress-induced hydrophobic peptide [*Populus trichocarpa*]	0.2422
PtpAffx.1159.1.S1_at	B9SKH2	Pectinesterase inhibitor, putative [*Ricinus communis*]	0.2328
PtpAffx.857.2.S1_at	B9SZU3	lactoylglutathione lyase, putative [*Ricinus communis*]	0.2294
Ptp.1037.2.S1_a_at	B9MW17	beta-ketoacyl-coa synthase family protein [*Populus trichocarpa*]	0.2249
PtpAffx.71561.1.A1_at	B9HRA6	Predicted protein[*Populus trichocarpa*]	0.2197
PtpAffx.210728.1.S1_at	Q6RVV4	short-chain dehydrogenase Tic32. [*Pisum sativum*]	0.2165
Ptp.3110.2.S1_a_at	B9HII6	1-cys peroxiredoxin [*Populus trichocarpa*]	0.2126
PtpAffx.17243.1.S1_at	B9HCZ1	predicted protein [*Populus trichocarpa*]	0.2067
PtpAffx.3298.2.S1_at	B9H859	predicted protein [*Populus trichocarpa*]	0.2034
Ptp.2835.1.S1_at	B9IMV1	predicted protein [*Populus trichocarpa*]	0.2008
PtpAffx.213305.1.S1_at	Q9LUF1	short-chain dehydrogenase/reductase family protein [*Arabidopsis thaliana*]	0.189
Ptp.1037.1.S1_s_at	B9MW20	beta-ketoacyl-coa synthase family protein[*Populus trichocarpa*]	0.1841
PtpAffx.217816.1.S1_s_at	Q9LTX3	Similarity to pyridoxamine 5-phosphate oxidase	0.1749
Ptp.4895.1.S1_x_at	Q9M4G9	putative ripening-related protein [*Vitis vinifera*]	0.1683
PtpAffx.159492.1.A1_at	B9GL20	predicted protein [*Populus trichocarpa*]	0.153

Table S3. *Cont.*

PtpAffx.17565.1.A1_at	Q94LH2	Putative calmodulin-like protein [Oryza sativa (japonica cultivar-group)]	0.1511
PtpAffx.209569.1.S1_at	O49710	26.5 kDa class P-related heat shock protein (HSP26.5-P) [*Arabidopsis thaliana*]	0.1462
Ptp.3826.1.A1_s_at	B9RTB7	Major pollen allergen Bet v 1-D/H, putative [*Ricinus communis*]	0.1395
PtpAffx.25008.1.S1_s_at	B9GL74	predicted protein [*Populus trichocarpa*]	0.0939
PtpAffx.9758.1.A1_at	B9RXT8	DUF26 domain-containing protein 1 precursor, putative [*Ricinus communis*]	0.0564

Table S4. Primer sequences for the real-time PCR.

Primer name	Forward primer	Reverse primer
Actin	GCTGGGTTTGCTGGGGAT	TCTGTAAGGAGGACTGGGTGCT
tubulin	GCACCAACTTGTTGAGAATGC	TTTCAACTGACCAGGGAACC
PtpAffx.95130.1.A1_s_at	GTTATGGTTGCTGGGGTGAGT	CTTGTTTGCATGGTTTTGGAG
Ptp.7096.1.S1_x_at	ACCAACAGATCCTTACGAGAGAG	CAAACCAACAAGTGAAGAGTCCAT
PtpAffx.213179.1.S1_a_at	ATGCTGTGTTAACTGCCTACGC	TTTGAACCCTCTGCCACTCTCT
PtpAffx.160390.3.A1_s_at	TTGCTTCCTTCCTCTGTCCATA	AGTCCCTGCTCCAAAACTTCTT
Ptp.5150.1.S1_s_at	TAGGAGGGTAGGTGAGCGGAAT	TAAATATAAAAGGAAAGGCGGGA
PtpAffx.857.2.S1_at	TGCAAGAAATGGGAATGAGGTAT	TTGAATGGGCAGGATGACAGAG
PtpAffx.200885.1.S1_x_at	CAGAAATGGGGCAGGGTTAT	GGGGTACTCCACGAAGAAGG
PtpAffx.125908.1.A1_at	ATGCTCTTTGTTTCCTTCCAG	AAGTGGTTGGTCTGCTTGTG
PtpAffx.35408.1.S1_at	GTATCGGAGGCGTTTCTTACA	TCTTTTCCTGCTCTGTTGCTT
PtpAffx.113991.1.A1_at	GGTCCAAATGACAGTCCCTAT	CAAGCAGATGTTCCCGTTAC
PtpAffx.225317.1.S1_at	ACCTTTCGTTTGGCTTGTAGA	TAGCTCGGTTGAATGGAGTC
PtpAffx.308.4.A1_at	TGGTTACTTATGCTTCAGTGCTC	CATGTTGCTTCAAGAAATCCC
PtpAffx.39778.1.A1_at	CAGCATTTGTGAAGGTCTGC	GTGAAGGAACTGAACATTTTGG
Ptp.127.1.S1_s_at	TGAAGCTGCTGGACTTTACG	GATCCATTATACCCTTCCCTCT
Ptp.128.1.S1_at	TTCCTTCACCATAGGCAACTG	GGACATCTCCGACGAGCATC

Table S5. Primer sequences for the real-time PCR.

Primer Name	Primer Sequence	Distance from Border
GSPL1	CTTGATTTGGGTGATGGTTCACG	332 bp
GSPL2	GGGATTTTGCCGATTTCGGAACCA	178 bp
GSPL3	ACCGCTTGCTGCAACTCTCTCAG	112 bp
GSPR1	TGCTAGCTGATAGTGACCTTAGGC	219 bp
GSPR2	AGTGGCTCCTTCAACGTTGCGGTTC	123 bp
GSPR3	TGACTCCCTTAATTCTCCGCTCATG	43 bp

© 2012 by the authors; licensee MDPI, Basel, Switzerland. This article is an open access article distributed under the terms and conditions of the Creative Commons Attribution license (http://creativecommons.org/licenses/by/3.0/).
